# Cell-Specific Single Viral Vector CRISPR/Cas9 Editing and Genetically Encoded Tool Delivery in the Central and Peripheral Nervous Systems

**DOI:** 10.1523/ENEURO.0438-23.2024

**Published:** 2024-07-03

**Authors:** Jamie C. Moffa, India N. Bland, Jessica R. Tooley, Vani Kalyanaraman, Monique Heitmeier, Meaghan C. Creed, Bryan A. Copits

**Affiliations:** ^1^Washington University Pain Center, Department of Anesthesiology, Washington University School of Medicine, St. Louis, Missouri 63110; ^2^Washington University Medical Scientist Training Program, Washington University School of Medicine, St. Louis, Missouri 63110; ^3^Washington University Division of Biological and Behavioral Sciences, Washington University School of Medicine, St. Louis, Missouri 63110; ^4^Departments of Neuroscience, Psychiatry, and Biomedical Engineering, Washington University School of Medicine, St. Louis, Missouri 63110

**Keywords:** CRISPR/Cas9, gene editing, imaging, optogenetics, photometry, tool

## Abstract

CRISPR/Cas9 gene editing represents an exciting avenue to study genes of unknown function and can be combined with genetically encoded tools such as fluorescent proteins, channelrhodopsins, DREADDs, and various biosensors to more deeply probe the function of these genes in different cell types. However, current strategies to also manipulate or visualize edited cells are challenging due to the large size of Cas9 proteins and the limited packaging capacity of adeno-associated viruses (AAVs). To overcome these constraints, we developed an alternative gene editing strategy using a single AAV vector and mouse lines that express Cre-dependent Cas9 to achieve efficient cell-type specific editing across the nervous system. Expressing Cre-dependent Cas9 from a genomic locus affords space to package guide RNAs for gene editing together with Cre-dependent, genetically encoded tools to manipulate, map, or monitor neurons using a single virus. We validated this strategy with three common tools in neuroscience: ChRonos, a channelrhodopsin, for studying synaptic transmission using optogenetics, GCaMP8f for recording Ca^2+^ transients using photometry, and mCherry for tracing axonal projections. We tested these tools in multiple brain regions and cell types, including GABAergic neurons in the nucleus accumbens, glutamatergic neurons projecting from the ventral pallidum to the lateral habenula, dopaminergic neurons in the ventral tegmental area, and proprioceptive neurons in the periphery. This flexible approach could help identify and test the function of novel genes affecting synaptic transmission, circuit activity, or morphology with a single viral injection.

## Graphical abstract

**Figure:**
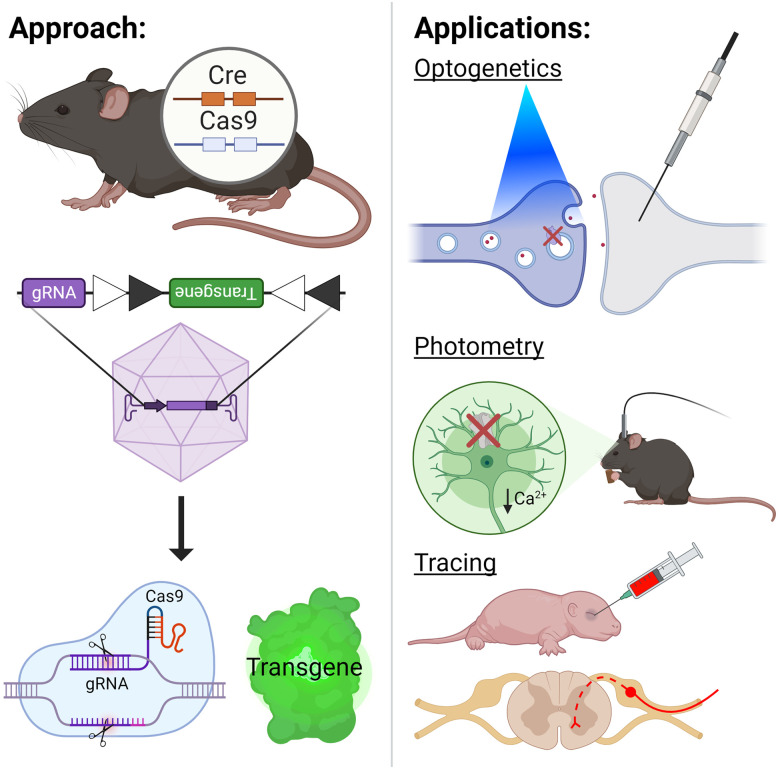
Visual Overview

## Significance Statement

Our CRISPR/Cas9 approach is the first to use a single vector to both knockdown genes of interest and express tools to monitor, map, and manipulate neurons. We demonstrate its utility in the central nervous system and describe the first systemic CRISPR/Cas9 gene editing with coexpressed reporters in the peripheral nervous system. Our approach fills a significant gap in the neuronal gene editing toolkit, allowing high-throughput study of genes of unknown function in the nervous system, and has broad utility for loss-of-function studies in other biological fields. This tool has great translational potential: it can be used to screen risk factor genes identified through genome-wide association studies or knockdown native gene expression and reintroduce mutant variants identified in clinical settings.

## Introduction

Advances in genetic targeting have revealed how specific genes influence normal and pathological nervous system functions. Combining these manipulations with genetically encoded tools provides powerful approaches to understanding how specific genes regulate synaptic, circuit, and behavioral phenotypes. Fluorescent proteins permit visualization of cellular structures (Tashiro et al., 2006; Young et al., 2008; Zhang et al., 2011; Chen et al., 2017; Zhang et al., 2022; Meltzer et al., 2023), while calcium sensors enable optical recording of neuronal activity (McQuillan et al., 2022; Zhou et al., 2022; Brandner et al., 2023). Opsins and chemogenetic tools can manipulate the activity of defined cell types to better understand their function (Stuber et al., 2010; Rapanelli et al., 2017; Hadjas et al., 2020; Garrido et al., 2022).

CRISPR/Cas9 gene editing has emerged as an effective method to knock out genes in a cell-type–specific manner. It uses bacterial Cas9 proteins, which complex with short guide RNAs (gRNA) to target specific DNA regions and cause a double-stranded break (DSB; Jinek et al., 2012; Jiang and Doudna, 2017; Kalamakis and Platt, 2023). DSB repair via nonhomologous end joining (NHEJ) often results in frame-shifting mutations generating a premature stop codon. Successful CRISPR/Cas9 editing has been demonstrated for multiple genes across diverse brain regions and cell types using viral vectors containing gRNA and Cre-dependent Cas9 injected into Cre-driver mice (Swiech et al., 2015; Hunker et al., 2020; Castro et al., 2021; Fellinger et al., 2021; Li et al., 2022; Liu et al., 2022; Gunduz-Cinar et al., 2023; Soden et al., 2023). However, this strategy requires the injection of two AAV vectors to achieve both editing and transgene expression. This is because Cas enzymes are large; only a few can be packaged into AAVs, which have a maximum capacity of ∼4.7 kb (Dong et al., 1996; Grieger and Samulski, 2005; Challis et al., 2019; Hunker et al., 2020). Thus, a second vector carrying a transgene must be coinjected. Injecting multiple viruses compounds inefficiencies in transduction, resulting in some edited neurons that are not labeled with the transgene and some unedited neurons that nevertheless contain the transgene. While AAV coinfection in some brain regions is high, cotransduction efficiency is lower in the cerebellum and striatum (Chan et al., 2017; Juarez et al., 2023; Maturana et al., 2023). Colabeling is also lower in tissues such as the retina and cochlea (Palfi et al., 2012; Akil, 2020). The mismatch between edited and transgene-expressing neurons could lead to data misinterpretations. Furthermore, systemic dual virus injection for editing in the peripheral nervous system (PNS) requires very high titers, making it prohibitively expensive. This approach is also limited by circulatory volume in young mice, severely curtailing the current utility of CRISPR/Cas9 gene editing with dual viruses in the PNS.

We designed and validated an alternative approach that addresses these limitations of two-vector CRISPR/Cas9 techniques. We first modified viral vectors with gRNA insertion sites (Swiech et al., 2015) to coexpress Cre-dependent transgenes to visualize, manipulate, and record target neurons. To circumvent the size limitations of AAVs, we crossed Cre-dependent Cas9 mouse lines (Platt et al., 2014; Chiou et al., 2015) to Cre-driver lines to enable cell-type–specific Cas9 expression. As this method only uses a single viral vector, the edited population of neurons fully overlaps with transgene expression. We validated this approach with the optogenetic tool ChRonos, GCaMP8f for recording Ca^2+^ transients, and mCherry for anatomical tracing. This approach can be used to screen and test the function of novel genes for effects on synaptic transmission, circuit activity, or morphology with a single viral injection.

## Materials and Methods

### Ethics approval

All procedures were conducted in accordance with National Institutes of Health guidelines and with approval from the Institutional Animal Care and Use Committee at Washington University in St. Louis.

### Viral vector generation

#### Cloning Cre-dependent transgene into pX552 empty vector

For all vectors, the pX552 vector (Addgene #60958; Swiech et al., 2015) with gRNA scaffold was used as a backbone.

To insert the DIO-mCherry construct into the pX552 vector, we cut pcDH-DIO-mCherry (Addgene #50462) with EF1a promoter with EcoRV and EcoR1. We cut pX552 with PspOM1 and cloned the pcDH-DIO-mCherry fragment into the opened pX552 vector. We then cut the new vector with EcoR1 and ran it on a gel to validate the insertion of the mCherry fragment.

To insert the DIO-ChRonos-GFP construct into the pX552 vector, we cut the previously generated pX552-EF1a-DIO-mCherry construct with SalI and fill and cut with HindIII. Cut pAAV-EF1a-FLEX-rChronos-GFP (Addgene #62725) with EcoR1 and fill and cut with HindIII.

To make DIO-GCamp8f, we cut pX552-DIO-synaptophysin-GFP with SpeI and EcoRV and synthesized a custom gene fragment (Azenta) for GCamp8f (Addgene #162379) lacking the original SapI site, to facilitate gRNA cloning.

##### Selecting gRNA candidates

To select candidate gRNA sequences (Hunker et al., 2020) for each target gene, we first used the National Center for Biotechnology Information (NCBI) Gene database to assess the exon structure and compare the splice variants of the target gene. The earliest exon shared among all splice variants was used as the target region for the gRNA search. We then obtained the coding sequence for the target exon from Ensembl.org. We used the CRISPOR web tool to identify suitable gRNA sequences within the target exon adjacent to *Streptococcus pyogenes* Cas9 protospacer adjacent motifs (spPAMs; Concordet and Haeussler, 2018). We pasted the exon coding sequence into the text box below “Step 1” on the CRISPOR home page, selected the “Mus musculus – Mouse (reference) – UCSC Dec. 2011 (mm10 = C57BL/6J)” under “Step 2,” and selected “20bp-NGG – SpCas9, SpCas9-HF1, eSpCas9 1.1” under “Step 3.” Two candidate sequences for each gene were selected that maximized the Doench ‘16 predicted efficiency (Doench et al., 2016) and MIT and CFD specificity scores while minimizing off-target matches within the mouse genome.

Sense and antisense candidate gRNA oligos were ordered from Genewiz/Azenta with the following modifications: (1) if the original gRNA candidate did not have a “G” residue at the 5′ end, a “G” was added to the 5′ end of the sense oligo, with a complimentary “C” added to the 3′ end of the antisense oligo. (2) To facilitate integration into Sap1 cut site sticky ends, the sequence “ACC” was added to the 5′ end of the sense oligo, and the sequence “AAC” was added to the 5′ end of the antisense oligo. All gRNA sequences are available in the material availability table (Table 1). After validating the Grin1 functional knockdown and submitting this construct to Addgene, we noticed a deletion of a “G” at position 5 in the Grin1 gRNA sequence. The sequence was changed from 5′-GCACGAGCAGATGTTCCGCG-3′ to 5′-GCACAGCAGATGTTCCGCG-3′. The sequences for the Grin1 gRNA in Table 1 and Figure 3 reflect this single base pair mutation. All other sequences are as intended.

**Table 1.** Material availability

Reagent type (species) or resource	Designation	Source or reference	Identifiers	Additional information
Strain, strain background (Rosa26-LSL-Cas9, C57Bl/6)	Rosa26Sortm1(CAG-cas9*,EGFP)Fezh	Jackson Laboratory; PMID 25263330 (Platt et al., 2014)	Jackson Laboratory #0126175	Mouse line with Cre-dependent Cas9 knocked in to the Rosa26 locus
Strain, strain background (H11-LSL-Cas9, C57Bl/6)	Igs2tm1(CAG-Cas9*)/Mmw	Jackson Laboratory (Chiou et al., 2015)	Jackson Laboratory #027632	Mouse line with Cre-dependent Cas9 knocked in to the Igs2 locus
Strain, strain background (Vgat-IRES-Cre, C57Bl/6)	Slc32a1tm2(cre)Lowl	Jackson Laboratory; PMID 21745644 (Vong et al., 2011)	Jackson Laboratory #028862	Mouse line with Cre recombinase knocked in to the Slc32a1 gene
Strain, strain background (Vglut2-IRES-Cre, C57Bl/6)	Slc17a6tm2(cre)Lowl	Jackson Laboratory; PMID 21745644 (Vong et al., 2011)	Jackson Laboratory #016963	Mouse line with Cre recombinase knocked in to theSlc17a6 gene
Strain, strain background (PV-IRES-Cre, C57Bl/6)	Pvalbtm1(cre)Arbr	Jackson Laboratory; PMID 15836427 (Hippenmeyer et al., 2005)	Jackson Laboratory #017320	Mouse line with Cre recombinase knocked in to the PV gene locus
Strain, strain background (TH-IRES-Cre, C57Bl/6)	Thtm1(cre)Te	MGI; PMID 15452869 (Lindeberg et al., 2004)	MGI #3056580	Mouse line with Cre recombinase knocked in to the TH gene locus
Genetic reagent (SapI)	SapI restriction enzyme	BioLabs	BioLabs #R0569S	SapI restriction endonuclease
Antibody	Mouse anti-VGAT	Synaptic Systems	Synaptic Systems #131011	Primary antibody against VGAT raised in mouse
Antibody	Donkey anti-mouse AF568	Invitrogen	Invitrogen #A10037	Secondary antibody against mouse IgG L chains with AF568 fluorophore
Recombinant DNA reagent	pX552: pAAV-U6sgRNA(SapI)_hSyn-GFP-KASH-bGH (SpGuide acceptor)	Feng Zhang (Swiech et al., 2015)	Addgene #60958	AAV plasmid for sgRNA cloning. GFP-KASH fusion facilitates FACS sorting of cells and nuclei
Recombinant DNA reagent	pAAV-EF1a-DIO-mCherry	Bryan Roth	Addgene #50462	Double floxed mCherry under the control of EF1a promoter
Recombinant DNA reagent	pAAV-EF1α-FLEX-rc[ChRonos-GFP]	PMID 24509633 (Klapoetke et al., 2014)	Addgene #62725	Cre-dependent fast channelrhodopsin ChRonos conjugated to GFP
Recombinant DNA reagent	pGP-AAV-syn-FLEX-jGCaMP8f-WPRE	DOI: 10.25378/janelia.13148243.v1 (Zhang et al., 2020)	Addgene #162379	AAV-mediated expression of ultrafast protein calcium sensor under the Syn promoter, Cre-dependent expression
Recombinant DNA reagent	pX552-EF1a-DIO-ChRonos-GFP(with gRNA scaffold)	This paper	Addgene #199582	Expresses ChRonos-GFP in a Cre-dependent manner
Recombinant DNA reagent	pX552-hsyn-DIO-GCaMP8f (with gRNA scaffold)	This paper	Addgene #199580	Expresses GCaMP8f in a Cre-dependent manner
Recombinant DNA reagent	pX552-EF1a-DIO-mCherry (with gRNA scaffold)	This paper	Addgene #199581	Expresses mCherry in a Cre-dependent manner
Sequence-based reagent	Vgat gRNA	This paper		Sense: 5′-ACCGCTGGGACTTGTTGGACACGG-3′ Antisense: 5′-AACCCGTGTCCAACAAGTCCCAGC-3′
Sequence-based reagent	VgatTTT gRNA	This paper		Sense: 5′-ACCGCTGGGACTTGTTGGACATTT-3′ Antisense: 5′-AACAAATGTCCAACAAGTCCCAGC-3′
Sequence-based reagent	Grin1 gRNA	This paper		Sense: 5′-ACCGCACAGCAGATGTTCCGCG-3′ Antisense: 5′-AACCGCGGAACATCTGCTGTGC-3′
Sequence-based reagent	Grin1TTT gRNA	This paper		Sense: 5′-ACCGCACGAGCAGATGTTCCTTT-3′ Antisense: 5′-AACAAAGGAACATCTGCTCGTGC-3′
Sequence-based reagent	Dicer gRNA	This paper		Sense: 5′-ACCGGACCCATTGGTGAGGAAGCA-3′ Antisense: 5′-AACTGCTTCCTCACCAATGGGTCC-3′
Sequence-based reagent	Dicer TTT gRNA	This paper		Sense: 5′-ACCGGACCCATTGGTGAGGAATTT-3′ Antisense: 5′-AACAAATTCCTCACCAATGGGTCC-3′
Sequence-based reagent	EGFP-O4 Probe	ACD	ACD #538851	ISH probe against EGFP used to label GCaMP8f RNA
Sequence-based reagent	TH Probe	ACD	ACD # 317621-C2	ISH probe against *Th*
Sequence-based reagent	*Pvalb* Probe	ACD	ACD #421931-C2	ISH probe against *PV*
Sequence-based reagent	*mCherry* Probe	ACD	ACD #431201	ISH probe against mCherry
Software, algorithm	CRISPOR	DOI: 10.1093/nar/gky354 (Concordet and Haeussler, 2018)	crispor.tefor.net	CRISPOR.org is a web tool for genome editing experiments with the CRISPR/Cas9 system
Software, algorithm	pyABF	https://pypi.org/project/pyabf/	pyABF 2.3.5	Python library for reading files in Axon Binary Format (ABF)
Software, algorithm	Igor Pro with NeuroMatic plug-in	Igor Pro: WaveMetrics NeuroMatic: PMID 29670519 (Rothman and Silver, 2018)		Software for ephys data analysis
Software, algorithm	Guided Photometry Analysis in Python (GuPPy)	PMID 34930955 (Venus N Sherathiya et al., 2021)		GuPPy, a Python toolbox for the analysis of fiber photometry data
Other	FED3	DOI: 10.7554/eLife.66173 (Matikainen-Ankney et al., 2021)	https://github.com/KravitzLabDevices/FED3	An open-source device for measuring food intake and operant behavior in rodent home cages

Key resources for reproduction of the methods and results described in this paper.

##### Cloning gRNA into pX552 gRNA site

Two micrograms of pX552 vector with SapI gRNA insertion site and Cre-dependent transgene were digested with 1:100 SapI enzyme (BioLabs #R0569S) in 1:10 CutSmart Buffer (BioLabs #B6004S) at 37°C for 1 h. Following the first incubation, 2 µl FastAP (Thermo Fisher Scientific #EF0651) was added to the solution, which was incubated at 37°C for 1 h and then 65°C for 20 min.

During the second digest, a 0.8% low-melt agarose gel with 50 µl combs was prepared. SYBR Safe dye (Invitrogen #S33102) was added to 5× DNA loading buffer at a ratio of 1 µl dye:50 µl loading buffer. Following incubation, 10.5 µl of 5× loading buffer/dye mixture was added to the 42 µl digest, which was run alongside a 1 kb ladder on 0.8% agarose gel at 80 V for 30 min. As a quality control, 2 µg of undigested vector was run alongside the digested product. After 30 min, gels were subjected to blue light and assessed for a ∼5 kb single band of linear DNA. This band was cut from the gel and digested using the Takara Gel Purification Kit (Takara #740609.50).

Meanwhile, 10 µl phosphorylation mix [1 µl 100 mM sense oligo, 1 µl 100 mM antisense oligo, 0.5 µl 25 mM ATP (BioLabs #P0756S), 1 µl 10× PNK T4 buffer (BioLabs #B0201S), 1 µl T4 PNK (BioLabs #M0201L), 5.5 µl dH_2_O] was prepared. gRNA oligos were phosphorylated according to the following protocol: (1) 37°C for 30 min, (2) 95°C for 5 min, and (3) cool to 4°C at 0.1°C/s. Phosphorylated oligos were diluted 1 µl oligo:49 µl dH_2_O. Following phosphorylation, 10 µl ligation mix was prepared [20 ng digested pX552 vector, 0.8 µl 1:50 diluted oligo mix, 5 µl 2×T7 Buffer (BioLabs #B0318S), 0.5 µl T7 DNA ligase (BioLabs #M0318S)] in dH_2_O. Vectors were ligated at room temperature for 30 min.

Following ligation, a 5 µl ligated vector was added to 25 µl Stbl3 cells (Invitrogen #C737303) and mixed by gently tapping. The mixture was incubated on ice for 30 min and then heat-shocked in a 42°C water bath for 45 s. After heat shock, cells were placed on ice for 2 min before adding 250 µl SOC medium (BioLabs #B9020S). The transformed cells in the medium were incubated in a shaker for 1 h at 37°C and 225 rpm. Fifty microliters of transformed cells in SOC medium were plated on agar plates with 1:1,000 carbenicillin (Sigma-Aldrich #C1389) and incubated overnight at 37°C. Following overnight incubation, three colonies of each construct were selected and incubated in 4 ml of LB medium (Sigma-Aldrich #L3022) with 1:1,000 carbenicillin overnight in a shaker at 37°C and 250 rpm. DNA from expanded colonies was purified using QIAprep Spin Miniprep Kit (Qiagen #27106), and concentration was determined using an Invitrogen Qubit fluorometer (Invitrogen #Q32857) and dsDNA Quantification Assay Kit (Invitrogen #Q32850). Samples were sent to Genewiz/Azenta to be sequenced from the U6 promoter. Sample sequences were assessed for integration of the correct gRNA sequence and any replication errors in the surrounding plasmid sequence. One sample of each construct was expanded overnight in 200 ml of LB with 1:1,000 carbenicillin at 37°C and 250 rpm. DNA from expanded colonies was purified using the Promega Midiprep Kit (Promega #A7640), and concentration was determined using a Qubit fluorometer. A sample of the final purified plasmid with gRNA insert was sent to Genewiz/Azenta for AAV-ITR sequencing of the entire packaging region.

### AAV production

#### HEK cell transfection

All AAV production protocols were performed as previously described (Challis et al., 2019). One day prior to transfection, HEK293T cells were split into three 15 cm tissue culture-treated Petri dishes (Corning #353025) and incubated in a 37°C tissue culture incubator with 5% CO_2_ overnight. PEI (Polysciences #239661) for transfection was prepared by dissolving 100 mg PEI in 310 ml of H_2_O and titrating the solution to pH 3. PEI solution was then incubated at 37°C for 4 h and shaken vigorously every half-hour. Finally, the solution was incubated overnight at 37°C, filter-purified the next day, and stored at −20°C.

For transfection, we first prepared a master mix of PEI in 1× DPBS (483 µl PEI per 1,000 µl total volume). Next, 1,000 µl of the master mix is required per 15 cm plate, so we prepared 3,000 µl PEI–PBS master mix. We then prepared a 3,000 µl DNA master mix in 1× DPBS (17.11 µg pX552, 68.45 µg AAV capsid, 34.23 µg pHelper). The 3,000 µl PEI–DPBS master mix was then added to the DNA–DPBS master mix dropwise, mixed by inverting 5×, incubated at room temperature for no >10 min, and inverted 5× again. In addition, 2,000 µl of the combined mixture was added to each plate of HEK cells, and the plates were returned to the incubator for 72 h.

#### HEK cell harvesting and stock solution preparation

After 72 h, transfected HEK cells were harvested by pouring off the growth media and adding 5 ml of DBPS per plate, and then HEK cells were detached using a cell scraper. Cells suspended in DPBS from each of the three plates were transferred to a 50 ml conical tube and centrifuged at 2,000 × *g* at room temperature for 15 min. The supernatant was discarded, and cell pellets were stored at −80°C overnight.

Next, we prepared solutions for AAV extraction. Lysis buffer (150 mM NaCl, 50 mM Tris base) was prepared in ddH_2_O and titrated to pH 8.5 using 12N HCl. A stock solution of 5 M NaCl in ddH_2_O and 5× PBS-MK (1 mM MgCl_2_, 2.5 mM KCl in 5× PBS) was also prepared. Finally, 15, 25, 40, and 60% iodixanol (IOD) stock solutions were prepared (**15% IOD,** 30 ml of 60% IOD, 24 ml of 5 M NaCl, 24 ml of 5× PBS-MK, 42 ml of ddH_2_O; **25% IOD,** 33 ml of 60% IOD, 16 ml of 5× PBS-MK, 0.2 ml of phenol red, 30.8 ml of ddH_2_O; **40% IOD,** 80 ml of 60% IOD, 24 ml of 5× PBS-MK, 16 ml of ddH_2_O; **60% IOD,** 80 ml of 60% IOD, 0.2 ml of phenol red).

##### AAV extraction and purification

The next day, frozen HEK cell pellets were thawed in a 37°C water bath. Five milliliters of lysis buffer were added to the thawed pellet and vortexed until the pellet was completely resuspended. The lysis buffer suspension was then subjected to three freeze-thaw cycles at −80 and 37°C, respectively. After the final thaw, 14.46 µl of 1 M MgCl2 and 5 µl Benzonase were added to the solution, which was then vortexed and incubated at 37°C for 30 min. Following incubation, the solution was vortexed again and centrifuged at 350 × *g* at 4°C for 20 min.

During centrifugation, iodixanol gradients were poured into 29.9 ml OptiSeal ultracentrifuge tubes (Beckman Coulter #361625). Gradients were poured in the following order, (1) 7.5 ml of 15% IOD, (2) 5 ml of 25% IOD, (3) 7.5 ml of 40% IOD, and (4) 5 ml of 60% IOD, by pressing the tip of the serological pipette against the bottom of the ultracentrifuge tube and releasing the contents slowly, so as not to disrupt the gradient. Each subsequent solution was layered under the previous, and care was taken to avoid air bubbles, which can disrupt the gradient layers.

After centrifugation, the supernatant was collected from the 50 ml conical tubes using a 2 ml serological pipette and slowly layered on top of the iodixanol gradient to avoid mixing with the 15% layer. The pellet of HEK cell debris was discarded. After all supernatant was added to the tube, all ultracentrifuge tubes were weighed and balanced with additional lysis buffer as needed. Tubes were capped and dried before being placed in a prechilled Ti-70 rotor, ensuring the tubes were placed in a balanced configuration. Tube spacers were placed on top of each tube, and the gradients were centrifuged at 350,000 × *g* at 18°C for 1.5 h.

During ultracentrifugation, we prepared Cytiva Vivaspin 20 100 kDa MWCO concentrator tubes (Cytiva #28932363) to concentrate the final product. Five milliliters of 70% ethanol were added to each tube and centrifuged at 4,000 rpm for 5 min. After centrifugation, we discarded the ethanol and added 10 ml of ddH_2_O to the tube, inverted it five times, and discarded the water. Another 10 ml ddH2O was added to the tube and centrifuged at 4,000 rpm for 5 min, then discarded. After preparing the concentrator tubes, we prepared a stock solution of DPBS plus 1:1,000 pluronic detergent (Invitrogen #24040032) to prevent virus adhesion to the tube walls. We removed the plunger from a 10 ml syringe, attached a 0.22 µm filter (TPP #99722) to the end, and then added 10 ml of DPBS–pluronic mixture to the syringe barrel. We allowed the solution to drip via gravity into the prepared concentrator tubes, timed such that there was 2–5 ml of DPBS left in the syringe barrel by the time the iodixanol–virus solution was ready to be added.

After ultracentrifugation, tubes were removed from the rotor and placed in a tube rack. Tube caps were removed, and the virus was extracted from the 40/60% iodixanol layer through the side of the tube using an 18-gauge needle attached to a 10 ml syringe. The needle was inserted through the tube bevel-up just above the 60% layer. When approaching the 40/25% interface, the needle bevel was rotated down to avoid extracting the protein layer. In total, approximately 5–7 ml of iodixanol/virus mixture is extracted per virus preparation. The virus/iodixanol mixture was then layered below the DPBS–pluronic solution described above. We inserted the plungers back into the 10 ml syringes and pushed the iodixanol–virus solution through the filter and into the upper chamber of the concentrator tube. Concentrators were then centrifuged at 3,000 × *g* at room temperature for 8 min. We repeated centrifugation until 2–5 ml of iodixanol/virus mixture remained in the upper chamber. At that point, flow-through in the bottom chamber was discarded, and 13 ml DPBS–pluronic solution was added to the upper chamber and mixed via pipetting. We continued centrifugation at 3,000 × *g* at room temperature until 200–500 µl of DPBS–virus solution remained in the upper chamber. The virus was removed from the upper chamber of the concentrator and stored in low protein binding tubes (Eppendorf #022431064) at −80°C.

After purification, AAV stock concentration was determined using the Takara AAV Titration Kit (Takara #6233) and an Applied Biosystems 7500 Fast Real-Time PCR System.

### Model organisms

All procedures were conducted in accordance with the National Institutes of Health guidelines and with approval from the Institutional Animal Care and Use Committee at Washington University in St. Louis. All mice were group-housed and kept on a 12 h light/dark cycle unless otherwise specified. To generate transgenic lines with the cell-specific expression of SpCas9, homozygous Rosa26-LSL-Cas9 knock-in mice (Rosa26Sor^tm1(CAG-cas9*,EGFP)Fezh^, Jackson Laboratory #0126175; Platt et al., 2014) were crossed to following homozygous Cre lines: Vgat-IRES-Cre (Slc32a1^tm2(cre)Lowl^, Jackson Laboratory #028862; Vong et al., 2011), and Vglut2-IRES-Cre (Slc17a6^tm2(cre)Lowl^; Jackson Laboratory #016963; Vong et al., 2011). Homozygous H11-LSL-Cas9 knock-in mice (Igs2^tm1(CAG-Cas9*)/Mmw^; Jackson Laboratory #027632; Chiou et al., 2015) were crossed to homozygous TH-IRES-Cre (Th^tm1(cre)Te^, MGI 3056580; Lindeberg et al., 2004) or parvalbumin (PV)-IRES-Cre (Pvalb^tm1(cre)Arbr^; Jackson Laboratory #017320; Hippenmeyer et al., 2005). All lines were maintained on a C57Bl/6 background, and we used mice heterozygous for both Cas9 and Cre recombinase for all experiments. All animals were group-housed with *ad libitum* access to food and water and maintained on a 12 h light/dark cycle. We used both male and female mice for all experiments and did not observe any effects of sex in our analyses.

### Stereotaxic surgery

For all intracranial virus injections, mice were initially anesthetized in an induction chamber with 5% isoflurane (Piramal Critical Care #6679401710) in room air. Following induction, their heads were shaved and disinfected with iodine and 70% ethanol, and they were secured in the stereotax using ear bars. We injected mice with 0.05 mg/kg of 1 mg/ml buprenorphine ER (ZooPharm) and a bolus of 1 ml 0.9% sterile saline (Pfizer #00409488850) at the beginning of the surgery. A midline incision was made on the scalp to expose the skull, and the head was balanced left to right and front to back using the bregma as the reference coordinate. All injections were made using a 32-gauge 1.0 µl Hamilton Neuros Syringe Point Style 4 (Hamilton #65458-02). The virus was infused at a rate of 100 nl/min, and the syringe was left in place for 10 min after infusion.

#### NAc

We injected 6–8-week-old Vgat-IRES-Cre/Rosa26-LSL-Cas9 mice with 150 nl of AAV5-Vgat-DIO-ChRonos-GFP (active) or AAV5-VgatTTT-DIO-ChRonos-GFP (control) at three coordinates along the dorsoventral axis: A/P, +1.7 mm; M/L, 0.8 mm (left); and D/V, −4.65, −4.5, and −4.4 mm, for a total of 450 nl virus injected per animal. Both control and active virus were titered to 1 × 10^12^ vg/ml.

#### Ventral pallidum

We injected 6–8-week-old Vglut2-IRES-Cre/Rosa26-LSL-Cas9 mice with 500 nl of the above Vgat or VgatTTT viruses at the following coordinates: A/P, +0.16 mm; M/L, 1.5 mm (left); and D/V, −4.6 mm.

#### VTA

We injected 6–8-week-old TH-IRES-Cre/H11-LSL-Cas9 mice with 1,000 nL of either AAV9-Grin1-DIO-GCaMP8f (active) or AAV9-Grin1TTT-DIO-GCaMP8f (control) at the following coordinates: A/P, −3.1 mm; M/L, 0.5 mm; and D/V, −4.5 mm. For animals used for electrophysiology, injections were performed unilaterally on the left side. For animals used for fiber photometry, the skull was scored before virus injection. Injections were performed bilaterally, and a fiber-optic probe (Doric #B28044015) was inserted unilaterally on the left side during the same surgery at A/P, −3.1 mm; M/L, 0.5 mm; and D/V, −4.55 mm. Both control and active virus were titered to 4 × 10^11^ vg/ml. For these animals, the fiber optic was secured in place first using C&B Metabond (Parkell #S380) and then Jet Denture Repair Acrylic (Lang Dental #1223).

### Retro-orbital AAV injection

To target peripheral proprioceptive neurons, we injected p0-1 PV-IRES-Cre/H11-LSL-Cas9 mice retro-orbitally with 1 × 10^12^ vg of either PHP.S-pX552-Dicer-DIO-mCherry (active) or PHP.S-pX552-DicerTTT-DIO-mCherry (control; packaged by UNC Neuroscience Center/BRAIN Initiative NeuroTools Core). Injections were done using a BD Ultra-Fine insulin syringe (BD #328440) following a previously established protocol (Yardeni et al., 2011).

### Electrophysiology

#### Acute slice preparation

Brain slices for electrophysiology recordings were prepared using a protective cutting and recovery method (Ting et al., 2014; Copits et al., 2021). For NAc recordings, 6 weeks after viral injections, mice were deeply anesthetized with ketamine and xylazine and transcardially perfused with cold oxygenated NMDG-aCSF containing the following (in mM): 93 N-methyl-D-glucamine, 2.5 KCl, 1.25 NaH_2_PO_4_, 30 NaHCO_3_, 20 HEPES, 25 glucose, 5 ascorbic acid, 2 thiourea, 3 sodium pyruvate, 0.5 CaCl_2_, and 5 MgCl_2_, at pH = 7.3 and 300–310 mOsm. For VTA recordings, 6 weeks after viral injections mice were deeply anesthetized with ketamine and xylazine and transcardially perfused with cold oxygenated choline aCSF containing the following (in mM): 93 choline chloride, 2.5 KCl, 1.25 NaH_2_PO_4_, 30 NaHCO_3_, 20 HEPES, 25 glucose, 5 ascorbic acid, 2 thiourea, 3 sodium pyruvate, 12 N-acetyl-L-cysteine, 0.5 CaCl_2_, and 5 MgCl_2_, at pH = 7.3 and 300–310 mOsm. Brains were rapidly removed, embedded in 2% low-melt agarose (Sigma-Aldrich, A0676), and 200-µm-thick horizontal (VTA) or 300-µm-thick coronal slices (NAc) were cut using a Compresstome (Precisionary Instruments, catalog #VF210–0Z). Slices were transferred to a recovery chamber containing oxygenated choline aCSF (VTA) or oxygenated NMDG-aCSF (NAc) at 32°C for 10 min before transfer to a holding chamber filled with oxygenated aCSF at 32°C containing the following (in mM): 124 NaCl, 2.5 KCl, 1.25 NaH_2_PO_4_, 24 NaHCO_3_, 5 HEPES, 12.5 glucose, 2 CaCl_2_, and 1 MgCl_2_, at pH = 7.3 and 300–310 mOsm. Slices were maintained in the dark at room temperature and allowed to recover >1 h before recording.

#### NAc IPSC recordings

Slices were transferred to the chamber of an upright microscope (SliceScope, Scientifica), perfused with room temperature oxygenated aCSF described above at ∼2 ml/min and visualized using IR-DIC microscopy. Evoked inhibitory postsynaptic currents (IPSCs) were recorded at 0 mV from GFP-negative cells of the nucleus accumbens in a field of ChRonos+ terminals from adjacent neurons. Whole-cell recordings were made using 4–5 MΩ pipettes filled with Cs^+^ internal solution, containing the following (in mM): 110 cesium gluconate, 8 tetraethylammonium chloride, 3 QX-314 bromide, 1.1 EFTA, 0.1 CaCl_2_, 10 HEPES, 4 Mg-ATP, 0.4 Na_2_GTP, and 10 Na_2_phosphocreatine, at a pH of 7.28 with CsOH and 292 mOsm. IPSCs were pharmacologically isolated with 10 µM NBQX and 50 µM D-APV (both from Hello Bio). Recordings were performed using the pCLAMP 11 software (Molecular Devices) controlling a MultiClamp 700B amplifier. Data were digitized at 10 kHz and filtered at 3 kHz. Cells were discarded if the series resistance was >30 MΩ or changed >20%. Wide-field photostimulation was delivered through a 40× objective using custom LEDs coupled to the back fluorescence port of the microscope. LEDs were triggered by TTL pulses from the amplifier to an LED current controller (Thorlabs, DC4104). All light intensities were calibrated using a photodiode (Thorlabs, S121C) and power meter (Thorlabs, PM100D).

#### LHb evoked IPSC and EPSC recordings

Whole-cell patch-clamp recordings were made from coronal slices (220 µm) of the lateral habenula (LHb). Slices were prepared using a vibratome (Leica VT 2100) in an ice-cold cutting solution [containing the following (in mM): 0.5 CaCl_2_, 110 C_5_H1_4_CINO, 25 C_6_H_12_O_6_, 25 NaHCO_3_, 7 MgCl_2_, 11.6 C_6_H_8_O_6_, 3.1 C_3_H_3_NaO_3_, 2.5 KCl, and 1.25 NaH_2_PO_4_) and continuously bubbled with 95% O_2_ and 5% CO_2_. Slices were incubated at 32°C for 20 min in aCSF [containing the following (in mM): 119 NaCl, 2.5 KCl, 1.3 MgCl_2_, 2.5 CaCl_2_, 1.0 Na_2_HPO_4_, 26.2 NaHCO_3_, and 11 glucose] followed by storage at room temperature until electrophysiological recordings were performed. Slices were hemisected and superfused with aCSF at 30 ± 2°C. Neurons were recorded using borosilicate glass pipettes (4–5 MΩ resistance) pulled on a micropipette puller (Narishige PC-100) filled with cesium-based internal solution [containing the following (in mM): 135 cesium methanesulfonate, 10 potassium chloride, 10 HEPES, 1 magnesium chloride, 0.2 EGTA, 4 Mg-ATP, 0.3 GTP, and 20 phosphocreatine, at pH7.3 and 289 Osm]. Neurons were held at −55 mV to assess glutamatergic synaptic transmission and +10 mV to assess GABAergic synaptic transmission. Currents were amplified, filtered at 2 kHz, and digitized at 10 kHz using a MultiClamp 700B amplifier and Digidata 1550 (Molecular Devices). Clampex version 11.4 (Molecular Devices) was used for data acquisition. Series resistance was monitored using a hyperpolarizing step of −15 mV for 5 ms every 10 s; the cell was discarded if the series resistance changed by >15%. Neurons were visualized using an Olympus 560 upright microscope; field LED illumination (CoolLED) was used to visualize and stimulate channelrhodopsin-expressing terminals (473 nm, paired 4 ms light pulses, 50 ms interstimulus interval, 13.7–18.2 mW). In addition, 100 µM picrotoxin was washed on to abolish GABAergic transmission. All agents were purchased from Sigma Biosciences.

#### VTA NMDAR/AMPAR recordings

Recordings were performed as above for the NAc. GCaMP8f+ neurons were identified using epifluorescence, and AMPA and NMDAR currents were recorded at −70 and +40 mV, respectively. A bipolar stimulating electrode (FHC catalog #30202) connected to a stimulus isolator (World Precision Instruments catalog #A365) was placed ∼200 mm rostral to the recorded cell to deliver electrical stimulation (0.5 ms). EPSCs were pharmacologically isolated with 10 mM bicuculline and 100 mM picrotoxin (both from Hello Bio).

### Electrophysiology analysis

#### NAc evoked IPSC analysis

Evoked IPSC recordings were exported in Axon Binary File (.abf) format. We used the pyABF package (Harden, 2022) to build custom code to analyze the peak evoked IPSC amplitude and produce trial-by-trial and averaged IPSC traces (available on GitHub). For each cell, peak IPSC amplitudes from 3–10 consecutive sweeps were averaged, and cell averages for edited and control conditions were compared in GraphPad Prism 10.0 using a two-tailed unpaired nested *t* test.

#### Ventral pallidum evoked IPSC/EPSC analysis

Evoked IPSC and EPSC recordings were exported in Axon Binary File (.abf) format. As above, we used the pyABF package to build custom code to determine the average current amplitude for 20 ms around peak IPSC/EPSC deflection and produce trial-by-trial and averaged IPSC traces (available on GitHub). For each cell, the 20 ms average values for 10–50 consecutive sweeps were averaged, and the absolute value of the ratio of IPSC:EPSC amplitudes was calculated. Average EPSC amplitude and average IPSC:EPSC ratio were compared in GraphPad Prism 10.0 using a two-tailed, unpaired nested *t* test.

#### VTA evoked NMDAR/AMPAR analysis

AMPA and NMDAR currents were analyzed using Igor Pro software (WaveMetrics) with the NeuroMatic plug-in (Rothman and Silver, 2018). AMPAR currents were quantified as the average within 1 ms of the peak response. NMDAR currents were quantified 50 ms after the peak outward current. Currents represent the average amplitude from 10–15 consecutive sweeps recorded at 20 s intervals. Statistical comparisons were done using a two-tailed, nested *t* test with GraphPad software.

All recordings and analyses were performed by investigators blinded to the group.

### Pavlovian cue/reward task

The pavlovian cue/reward task was performed in a clear 6.75″ × 12.25″ × 9.875″ (W × L × H) plexiglass enclosure with a top-down camera monitoring mouse activity. For 2 weeks prior to the first recording session, mice were habituated to a reversed 12 h light/dark cycle. For 3 d prior to the first recording session, mice were acclimated to the chamber and to the fiber-optic cable attachment for 1 h/d starting at ZT13. No photometry recordings were made during this time. Between animals, the enclosure was cleaned with Clidox and water. Twelve hours prior to the first recording session (ZT1), food was removed from the animals’ home cages.

Animals were subjected to five 30-min-long recording sessions over the course of a week, occurring between ZT13-15. During sessions, a FED3 device (Matikainen-Ankney et al., 2021) delivered a 4,000 Hz, 200 ms tone followed 1–3 s later by a food reward pellet. The tone was coupled with a TTL pulse to Bonsai to allow for the alignment of photometry recording data with the tone cue. The mouse was freely able to take the pellet at will; reward acquisition was also coupled with a TTL pulse. Following reward acquisition, a variable 6–12 s interval preceded the tone cue for the next trial. Mice were not pretrained on this paradigm and learned it over the course of the five trial days. Between trial days, animals received food *ad libitum* until 12 h prior to the next day’s trial, at which point food was removed from their home cage.

### Fiber photometry setup and analysis

Fiber photometry signals were collected using a 2 m Doric fiber-optic cable (Doric #D20714022). A Plexon 473 nm LED driver was used to deliver a constant 2.7 W blue light stimulus via the cable, and GCaMP8f photons were collected by the same fiber optic, passed through a dichroic mirror, and recorded in Bonsai. No isosbestic control was used. The video was captured using a FLIR camera (Sony IMX273, 226 FPS). Photometry signal, video, mouse (X, Y) position, and tone and reward TTL signals were recorded and synchronized using Bonsai. The photometry signal data was output as a .csv and analyzed using Guided Photometry Analysis in Python (GuPPy; Sherathiya et al., 2021). Since no isosbestic control was used, GuPPy used a moving average to calculate a baseline fluorescence curve, accounting for photobleaching during the recording. To analyze the average GCaMP8f fluorescence response to cue and reward stimuli, cue and reward times were extracted from the raw data and input into GuPPy. The raw fluorescence signal was normalized by dividing the change in fluorescence by the baseline fluorescence (ΔF/F), and the epoch from 2 s before to 2 s after each tone cue or reward acquisition was analyzed to determine the peak GCaMP8f response to cue or reward, respectively. An example input parameters document is available on GitHub. Heatmaps were generated in Python by averaging the GCaMP8f signal from 2 s before to 2 s after the cue or reward from all trials on all days for each subject. Overall cue and reward GCaMP8f responses were compared in Prism, where columns represented control versus edited groups and subcolumns represented each subject. The peak Δ*F*/*F* response to cue or reward for all trials was entered for each subject, and the control versus edited responses were compared using an unpaired, two-tailed nested *t* test.

All photometry recordings and initial analysis in GuPPy were performed by investigators blinded to the group.

### Immunohistochemistry

#### VGAT

For VGAT immunohistochemistry (IHC) in the NAc, mice were perfused with 40 ml of chilled 1× PBS followed by 40 ml of chilled 4% PFA. Brains were dissected out and postfixed in 4% PFA overnight at 4°C. A Precisionary VF 310-0Z compresstome was used to cut 40-µm-thick coronal sections around the region containing the NAc. Sections were washed three times in 1× PBS and then incubated for 1 h in a blocking solution [0.3% Triton X-100 (Sigma-Aldrich), 5% normal donkey serum]. During incubation, a 1:1,000 solution of mouse anti-VGAT antibody (Synaptic Systems #131011) in a blocking solution was made. Slices were incubated in VGAT antibody solution overnight at 4°C. The next day, slices were washed three times in 1× PBS and then incubated for 2 h at 4°C in a 1:1,000 solution of AF-568 donkey anti-mouse secondary antibody (Invitrogen #A10037) in a blocking solution. Slices were washed three times in 1× PBS, mounted on a slide, and counterstained with DAPI (SouthernBiotech #010020).

### In situ hybridization

#### VTA

To verify the injection site, fiber placement, GCaMP8f/Th overlap in VTA sections and to assess *Grin1* nonsense-mediated decay (NMD), we performed in situ hybridization (ISH) using the RNAScope Multiplex Fluorescence v2.0 kit (ACD #323110). Mice were perfused with 40 ml of chilled 1× PBS followed by 40 ml of chilled 4% PFA. Brains were dissected out and postfixed in 4% PFA overnight at 4°C and then transferred to 30% sucrose in 1× PBS for overnight cryopreservation. Cryopreserved brains were frozen in OCT (Sakura Finetek #4583) at −80°C, and 20 µm sections were taken on a Leica CM1860 cryostat. Sections were immediately mounted on glass slides, dried at room temperature for 1 h, and then stored at −80°C until processing. ISH was performed according to the RNAscope Multiplex Fluorescent Reagent Kit v2 User Manual, following tissue pretreatment instructions for fixed-frozen tissue. To identify cells expressing GCaMP8f mRNA from the virus injection, we used the EGFP-O4 Probe in Channel 1 (ACD #538851). To label *Th-*expressing cells in the VTA, we used the *Th* Probe in Channel 2 (ACD # 317621-C2). To label *Grin1* transcripts, we used the *Grin1* probe in Channel 3 (ACD #431611-C3). We used Opal 520 reagent (Akoya #OP001001) to visualize EGFP-O4, Opal 570 reagent (Akoya #OP001003) to visualize *Grin1*, and Opal 650 Reagent (Akoya #OP001005) to visualize *Th*. After ISH, slices were coverslipped with DAPI mounting media.

#### DRG

For analysis of infection efficiency and to assess *Dicer1* NMD, DRG sections were dried, and ISH was performed using RNAscope, as above, with a probe against *PV* (ACD #421931-C2) to identify target DRG neurons, a probe against *mCherry* (ACD #431201) to identify infected neurons, and a probe against *Dicer* (ACD #409421-C3). The *PV* probe was tagged with Opal 650 reagent (Akoya #OP001005), the *mCherry* probe was tagged with Opal 570 reagent (Akoya #OP001003), and the *Dicer* probe was tagged with Opal 520 reagent (Akoya #OP001001).

#### NAc

For analysis of *Vgat* NMD, NAc sections were dried, and ISH was performed using RNAscope, as above, with a probe against *Gfp* (ACD #409971) to identify infected NAc neurons and a probe against *Vgat* (ACD #319191-C3). The *Gfp* probe was tagged with Opal 520 reagent (Akoya #OP001001), and the *Vgat* probe was tagged with Opal 650 reagent (Akoya #OP001005).

### Imaging

#### NAc imaging for VGAT puncta quantification

Experimenters were blinded to subject ID and condition during both imaging and analysis. Stained NAc sections were imaged on a Keyence BZ-X810 microscope at 60× magnification and high resolution with optical sectioning using a 10 µm 1D slit. DAPI was imaged with a blue dichroic filter (Chroma #49021) at 1/5 s exposure, GFP was imaged with a green dichroic filter (Chroma #49011) at 1/10 s exposure, and VGAT (AF568) was imaged with a red dichroic filter (Chroma #49008) at 1/3.5 s exposure for all sections across experiments with the same excitation intensity. All images were taken in areas with complete GFP expression to ensure an accurate comparison of VGAT puncta counts between images.

#### VTA imaging for virus expression and probe alignment

Stained VTA sections were imaged on a Keyence BZ-X810 microscope at 4× magnification. DAPI was imaged with a blue dichroic filter (Chroma #49021) at 1/2.5 s exposure, Opal 520 (GCaMP8f) was imaged with a green dichroic filter (Chroma #49011) at 1/3 s exposure, and Opal 650 (*Th*) was imaged with a far-red dichroic filter (Chroma #49006) at 3 s exposure. Mice with poor GCaMP8f expression were excluded from the analysis. Mice where fiber placement was >100 µm dorsal or lateral to GCaMP8f expression and those where fiber track was not located in the same slice as GCaMP8f expression were also excluded from the analysis.

#### Spinal cord

PV-Cre/Cas9 mice were perfused at p14 using 1× PBS followed by 4% PFA, as above. Following perfusion, animals were decapitated and postfixed in 4% PFA at 4°C overnight. Lumbar spinal cords and DRG were dissected out and postfixed overnight and then cryopreserved in 30% sucrose at 4°C. Spinal cords and DRG were frozen separately in OCT at −80°C; 40 µm sections of both spinal cord and DRG were taken using a Leica CM1860 cryostat at −20°C and mounted on separate glass slides. Spinal cord sections were counterstained with DAPI and imaged on a Keyence BZ-X810 microscope at 20× magnification, using optical sectioning with a 10 µm 1D slit. DAPI was imaged with a blue dichroic filter (Chroma #49021) at 1/10 s exposure, and mCherry was imaged with a red dichroic filter (Chroma #49008) at 1/10 s exposure. Images were stitched using BZ-X800 Analyzer, and stitched images were imported into FIJI for analysis.

#### DRG

DRG were imaged using a Keyence BZ-X810 microscope at high resolution and 40× magnification, using optical sectioning with a 10 µm 1D slit. DAPI was imaged with a blue dichroic filter (Chroma #49021) at 1/10 s exposure, Opal 570 (*mCherry*) was imaged with a red dichroic filter (Chroma #49304) at 1/3.5 s exposure, and Opal 650 (*PV*) was imaged with a far-red dichroic filter (Chroma #49006) at 1/1.2 s exposure.

#### NMD imaging

Images for NMD analysis were taken on a Leica confocal DMI8-CS microscope at 40× magnification with an HC PL APO CS2 oil immersion lens with a numerical aperture of 1.3. Images with 1,024 × 1,024 pixel resolution were captured at a scan speed of 400.

##### Vgat

The blue channel (DAPI) was excited using a Diode 405 nm laser, and wavelengths between 415–471 nm were captured. The laser intensity was set to 8.02% and the gain was set to 11.2. The green channel (*Gfp*) was excited using an OPSL 488 nm laser, and wavelengths between 493–550 nm were captured. The laser intensity was set at 15.01% and the gain was set to 3.5. The far-red channel (*Vgat*) was excited using a Diode 638 nm laser, and wavelengths between 644–850 nm were captured. The laser intensity was set at 6.01% and the gain was set to 2.7.

##### Grin1

The blue channel (DAPI) was excited using a 405 nm diode laser, and wavelengths between 415–450 nm were captured. The laser intensity was set to 2%, and the gain was set to 14.9. The green channel (*Gcamp8f*) was excited using a 488 nm OPSL laser, and wavelengths between 500–550 nm were captured. The laser intensity was set at 1.5%, and the gain was set to 2.5. The red channel (*Grin1*) was excited using a 561 nm DPSS laser, and wavelengths between 600–630 nm were captured. The laser intensity was set at 2%, and the gain was set to 19.2.

##### Dicer

The blue channel (DAPI) was excited using a 405 nm diode laser, and wavelengths between 415–450 nm were captured. The laser intensity was set to 2%, and the gain was set to 7.4. The green channel (*Dicer*) was excited using a 488 nm OPSL laser, and wavelengths between 500–550 nm were captured. The laser intensity was set at 8.5%, and the gain was set to 6. The red channel (*mCherry*) was excited using a 561 nm DPSS laser, and wavelengths between 600–630 nm were captured. The laser intensity was set at 2%, and the gain was set to 6.

### Image analysis

#### VGAT puncta

Red channel images were uploaded into FIJI (FIJI Is Just ImageJ) and analyzed as follows: first, pseudo-colored images were converted to 16-bit grayscale images. We then performed background subtraction with a rolling ball radius of 50 pixels. Images were converted to binary, using the threshold command such that all pixels with a value ≥9 were set to the maximum pixel value and all values <9 were set to 0. Puncta were counted using the “Analyze Particles” command, selecting for puncta between 0.001 and 0.1 in^2^ and circularity between 0 and 1. The number of puncta per image was compared between control and edited groups using an unpaired, two-tailed, nested *t* test in GraphPad Prism 10.0.

#### Spinal cord

For analysis of mCherry+ projections to the spinal cord dorsal horn, stitched red channel images were converted to 16-bit black and white images, and background subtraction was performed with a rolling ball radius of 50 pixels. ROIs were then drawn bilaterally around the point of fiber entry in the ventral horn, the intermediate zone (IZ), and the dorsal horn. We then calculated the fluorescence intensity for each ROI and normalized it to the area in µm^2^. Images were then binarized with a threshold of 4, and the binary images were used to calculate the %Area of mCherry+ fibers in each region. Fluorescence density and %Area values were averaged for each region in each subject and compared using an unpaired, two-tailed nested *t* test.

#### DRG

Red and far-red channel images (representing *mCherry*+ and *PV*+ cell bodies, respectively) were loaded into FIJI. Images were converted to 16-bit and background-subtracted using a rolling ball radius of 50 pixels. *mCherry+* and *PV+* cell bodies were identified manually, and the number of *mCherry+* cell bodies was divided by the number of *PV+* cell bodies to obtain the fraction of *PV+* neurons infected with virus. The fraction of *mCherry+/PV+* neurons in each section was averaged for each subject, and average control and edited infection efficiency were compared in GraphPad Prism 10.0 using an unpaired, two-tailed nested *t* test.

#### NMD

##### Vgat

Green channel (*Gfp*) images were loaded into Cellpose (Stringer et al., 2021). For segmentation, the estimated cell diameter was set to 40 µm, flow threshold was set to 0.4, cellprob threshold was set to 0, and stitch threshold was set to 0. The “cyto” model was used to generate initial ROIs, which were examined and modified by the researchers to ensure quality. ROIs were then exported for use in FIJI analysis. Far-red (*Vgat*) images were loaded into FIJI and converted to 16-bit and then background-subtracted as above. Fluorescence intensity was then measured in each ROI identified in Cellpose and normalized to the ROI area. Images were then binarized with a threshold of 25 and watershedded to increase delineation between puncta. Puncta were counted using the “Analyze Particles” command, selecting for puncta between 0.1–5 µm and circularity between 0–1. The number of puncta was normalized to the ROI area, and the %Area in each cell occupied by the identified puncta was also calculated. Fluorescence density/µm^2^, *Vgat* puncta/µm^2^, and *Vgat* puncta %Area were compared between Vgat-edited and TTT control groups using a two-tailed, nested *t* test.

##### Grin1

Green channel (*Gcamp8f*) images were loaded into Cellpose, and cells were identified as above. Red channel (*Grin1*) images were loaded into FIJI and preprocessed as above. Fluorescence intensity was measured in each ROI as above, and images were binarized with a threshold of 15 and watershedded. Puncta/µm^2^ and puncta %Area were calculated as above and all metrics were compared between Vgat-edited and TTT control groups using a two-tailed, nested *t* test.

##### Dicer

Red channel (*mCherry*) and green channel (*Dicer*) images were loaded into FIJI. Red channel images were used to identify ROIs manually. Green channel images were preprocessed, and fluorescence intensity was measured in each ROI as above. Images were then binarized with a threshold of 30 and watershedded. Puncta/µm^2^ and puncta %Area were calculated as above and all metrics were compared between Vgat-edited and TTT control groups using a two-tailed, nested *t* test.

All tissue processing, imaging, and initial analysis in FIJI were performed by experimenters blinded to the group.

### Indel analysis

Twenty microliters of cultured Neuro2a (N2A) cells were nucleofected on a Lonza 4D-Nucleofector with 500 µg of Cas9 expression plasmid and 1 µl of active Vgat or TTT control plasmid. Opti-MEM was used as the nucleofection solution. Following nucleofection, cells were seeded into 500 µl of recovery media in 24-well plates for 72 h. After 72 h, cells were harvested and underwent 35 cycles of PCR at 60°C in a SuperFi II machine with next-generation sequencing (NGS) genotyping primers. NGS was performed with Illumina, and reads were analyzed for indel fraction using the CRISpy v2 package (Connelly and Pruett-Miller, 2019).

### Statistics

Experimenters were blinded during data collection and processing. All data were analyzed in GraphPad Prism 10.0. For all electrophysiology imaging, and fiber photometry experiments, control and edited groups were compared using an unpaired, two-tailed, nested *t* test. All analysis code and GuPPy input parameters are available at https://github.com/jamiecm17/CRISPR-Cas9-Paper. All data are reported as mean ± SEM.

## Results

### Combining CRISPR/Cas9 with optogenetics to study synaptic physiology

**Figure 1.**
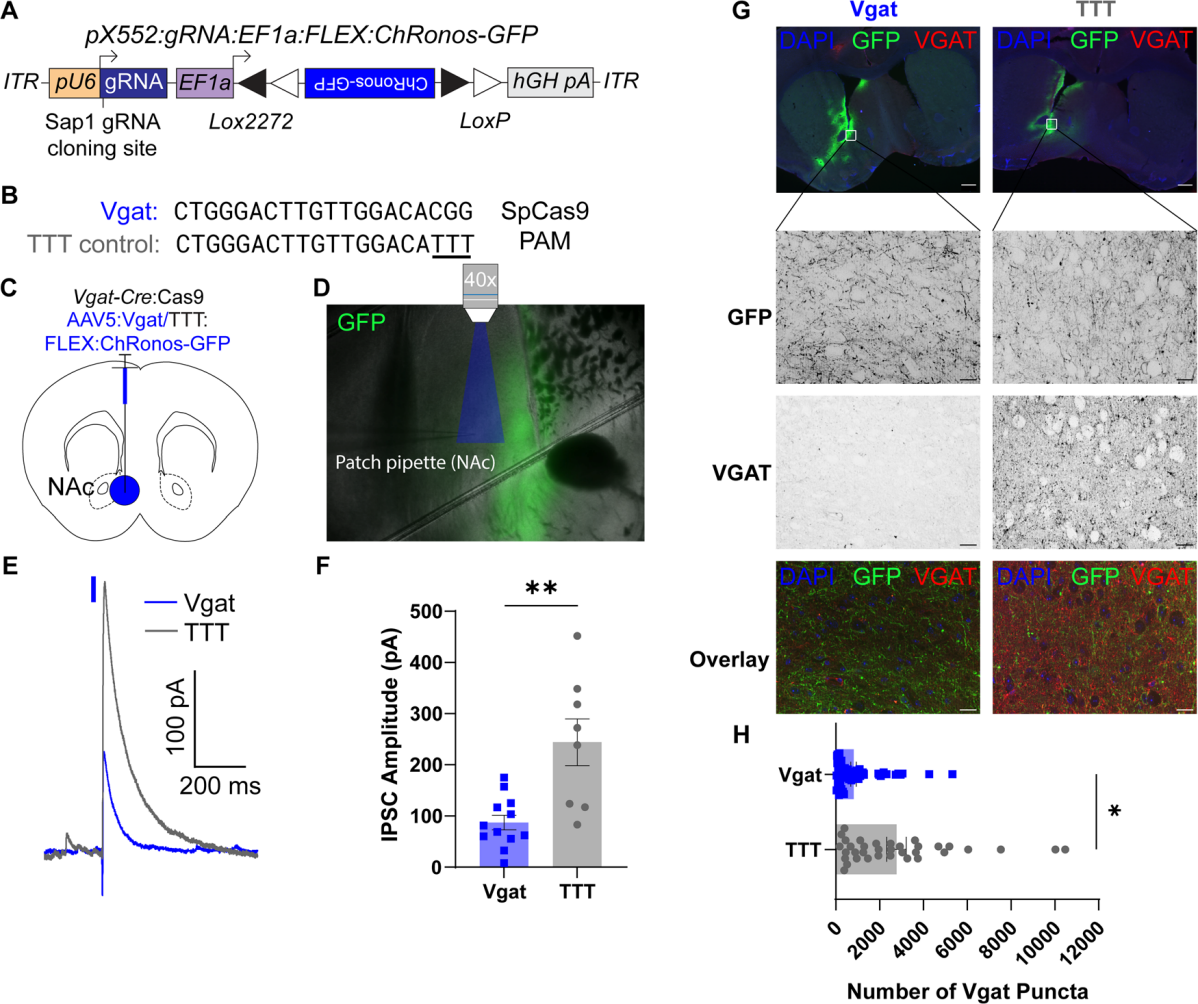
ChRonos-GFP coupled with *Vgat* editing demonstrates reduced inhibitory synapse function. ***A***, Schematic of pX552 viral vector with Cre-dependent ChRonos-GFP transgene and Vgat gRNA for gene editing. ***B***, Sequences of gRNA targeting *Vgat* (top) and modified TTT control (bottom). ***C***, Diagram of virus injection into the NAc of 6–8-week-old *Vgat*-Cre:Cas9 mice. ***D***, Representative image of slice electrophysiology setup, 4× magnification. The area of blue light stimulation of ChRonos shown in blue. ChRonos-GFP expression shown in green. ***E***, Representative oIPSC traces from *Vgat-*edited (blue) and TTT control (gray) neurons. Traces aligned to light pulse onset (blue rectangle). The representative edited IPSC trace is an average of five consecutive sweeps; the representative control IPSC trace is an average of four consecutive sweeps. ***F***, Quantification of evoked IPSC amplitude. Each point represents the average peak inhibitory current amplitude after blue light stimulus from 3–10 consecutive sweeps. The average Vgat IPSC amplitude was 87.28 ± 49.63 pA (*n *= 12 cells from 3 animals), and the average TTT IPSC amplitude was 244.3 ± 45.64 pA (*n* = 8 cells from 2 animals; *p* = 0.0012**^a^). ***G***, Representative VGAT IHC images for Vgat-edited (left) and TTT (right) conditions. Top, 10× images showing GFP expression for virus injection distribution (green), VGAT puncta labeling (red), and DAPI counterstain (blue). Second row, 60× images showing GFP expression in the NAc of Vgat (left) and TTT (right) slices. Third row, 60× images showing VGAT labeling in Vgat-edited (left) and TTT (right) slices. Bottom, Overlay of GFP (green), VGAT (red), and DAPI (blue). 10× scale bars = 100 µm; 60× scale bars = 20 µm. ***H***, Quantification of the number of VGAT puncta in Vgat-edited (top) or TTT (bottom) sections. Each point represents the number of VGAT puncta in one 241.56 × 181.17 µm NAc section imaged at 60×. The average number of puncta in Vgat-edited sections was 813.5 ± 124.3 puncta (*n* = 70 sections from 6 animals); the average number of puncta in TTT sections was 2,774 ± 444.4 puncta (*n* = 35 sections from 4 animals; *p* = 0.049*^b^). All comparisons were done using a two-tailed, nested *t* test. All data are reported as mean ± SEM. See Extended Data Figure 1-1 for more details.

To test the utility of our single-vector approach for transgene expression and CRISPR/Cas9 editing, we first focused on the vesicular GABA transporter (*Slc32a1* or *Vgat*) that packages GABA into synaptic vesicles, where effective editing should reduce the amplitude of optically evoked inhibitory postsynaptic currents (oIPSCs). To do this, we cloned the Cre-dependent fast channelrhodopsin ChRonos-GFP (Klapoetke et al., 2014) into an empty pX552 gRNA viral vector (Swiech et al., 2015; Fig. 1*A*). We next used the bioinformatics database CRISPOR (Concordet and Haeussler, 2018) to design a gRNA against *Vgat* and a nearly identical control gRNA that differed from the active construct by three thymidine residues near the protoadjacent spacer motif (PAM; Hunker et al., 2020; Fig. 1*B*). We inserted either the active or control gRNA into the *SapI* cloning site of the pX552:U6:gRNA:EF1a:FLEx:ChRonos-GFP construct and packaged these into AAV5 viruses to efficiently target CNS neurons. To selectively express Cas9 in GABAergic neurons, we crossed *Vgat*-Cre mice (Vong et al., 2011) to a Cre-dependent Cas9 line (Platt et al., 2014).

10.1523/ENEURO.0438-23.2024.f1-1 Figure 1-1 Efficiency of NAc *Vgat* editing and nonsense-mediated decay. (**A**) Quantification of the percentage of insertions, deletions, and single nucleotide polymorphisms (SNPs) in Neuro2a cells nucleofected with active Vgat plasmid (top) or TTT control (bottom). In the Vgat sample, edits were present in 69.9% of reads, with deletions making up 41.8% of reads and insertions making up 27.7% of reads. SNPs were present in 0.4% of reads. The most common single edit was a +1-nucleotide insertion, which occurred in 556 of 2177 total reads (25.5%). In the TTT control group, edits were present in only 1.2% of reads, with SNPs making up 0.9% of reads and deletions making up 0.3% of reads. There were no insertions. (**B**) Representative *in situ* hybridization images for Vgat (top) and TTT control (bottom) injections into the nucleus accumbens of adult *Vgat-*Cre;igs-Cas9 mice. **Left:** 40x images staining for *GFP* mRNA. **Middle:** 40x images staining for *Vgat* mRNA. **Right:** Merged images showing *GFP* in green, *Vgat* in magenta, and DAPI in cyan. Scale bars=20µm. (**C**) Quantification of the area-normalized fluorescence intensity of *Vgat* mRNA transcripts in Vgat edited vs. TTT control tissue. The average fluorescence intensity of the Vgat group was 173.4±25.9 AU/µm^2^ (n=4649 cells from 3 animals); the average for the TTT control group was 176.7±37.3 AU/µm^2^ (n=4365 cells from 3 animals) (p=0.95; ns^t^). (**D**) Quantification of the number of *Vgat* mRNA puncta per µm^2^ in Vgat edited vs. TTT control tissue. The average number of puncta for the Vgat group was 0.09±0.02 puncta/µm^2^ (n=4649 cells from 3 animals); the average for the TTT control group was 0.07±0.004 puncta/µm^2^ (n=4365 cells from 3 animals) (p=0.28; ns^u^). (**E**) Quantification of the *Vgat* mRNA percent area per cell. The average %Area/Cell for the Vgat group was 8.2±1.5% (n=4649 cells from 3 animals); the average for the TTT control group was 6.9±0.3% (n=4365 cells from 3 animals) (p=0.42; ns^v^). All comparisons were done using a 2-tailed, nested t-test. All data are reported as mean±SEM. Download Figure 1-1, TIF file.

To validate the editing efficiency of our construct, we conucleofected Neuro2a cells with either our active Vgat plasmid or TTT control and a Cas9 expression vector. After 72 h, cells underwent PCR and next-generation sequencing (NGS) at the cut site to identify the fraction of cells expressing NHEJ-mediated insertions or deletions. We found that the active Vgat construct had a mutagenesis efficiency of 69.9%; meanwhile, the TTT control plasmid had almost no activity (1.2%) at the indicated cut site (Extended Data Fig. 1-1*A*). We further tested whether our Vgat gRNA construct had off-target activity in other genes. We identified two potential off-target sites in the exons of Onecut1 and Tmem119 using CRISPOR (Concordet and Haeussler, 2018). We nucleofected Neuro2a cells as above and performed NGS at the off-target sites in Onecut1 and Tmem119. We found minimal off-target activity at each gene (Onecut1, 2.1%; Tmem119, 0.5%), indicating that our Vgat gRNA construct has minimal off-target activity in exons other than the target site (Extended Data Table 1-2). To assess whether our construct induces nonsense-mediated decay (NMD) of mutated *Vgat* mRNA, we injected either the active Vgat or TTT control virus into the NAc of 6–8-week-old *Vgat*-Cre/lsl-Cas9 mice. After 6 weeks, we performed in situ hybridization for *Gfp* and *Vgat* mRNA (Extended Data Fig. 1-1*B*). We failed to identify a significant reduction in the area-normalized *Vgat* fluorescence density, number of *Vgat* puncta/µm^2^, or *Vgat* puncta %Area per infected cell (Extended Data Fig. 1-1*C–E*).

10.1523/ENEURO.0438-23.2024.t1-2 Table 1-2 Vgat gRNA off-target editing at 2 potential off-target sites located in the exons of Onecut1 (row 2) and Tmem119 (row 3). Download Table 1-2, XLSX file.

To test whether our approach could induce functional reductions in optically evoked IPSCs in vivo, we injected 6–8-week-old *Vgat*-Cre/lsl-Cas9 mice with control or active virus in the nucleus accumbens (NAc; Fig. 1*C*). We chose the NAc because it contains a high density of locally-projecting GABAergic neurons to test the efficacy of VGAT manipulations.

After 6 weeks, we performed whole-cell patch-clamp recordings from acute slices of the NAc. We recorded pharmacologically isolated oIPSCs at 0 mV by stimulating ChRonos with blue light (1 ms, 1 mW/mm^2^; Fig. 1*D*). We found that oIPSC amplitudes in neurons from mice injected with the active virus were significantly reduced compared with control (Fig. 1*E,F*).

To validate that our viral vector reduces VGAT expression in GABAergic NAc neurons, we injected either the active or control virus into the nucleus accumbens (NAc) of adult *Vgat*-Cre/lsl-Cas9 mice, as above. After 6 weeks to allow for transgene expression and editing, we analyzed VGAT puncta using immunohistochemistry (IHC) and found that mice injected with active gRNA had significantly fewer VGAT puncta in GFP+ regions of the NAc, compared with control mice (Fig. 1*G,H*). These data demonstrate that our single-vector approach can be used to edit VGAT, visualize edited neurons, and investigate functional changes in edited neurons using optogenetics.

While our results targeting GABAergic neurons in the NAc suggest efficient editing, it is possible that the variation in oIPSC amplitudes could be due to differences in viral injection placement or efficiency, leading to optogenetic activation of different numbers of presynaptic terminals between the two groups. To rule out this possibility, we next targeted *Vglut2*+ neurons that project from the ventral pallidum (VP) to the lateral habenula (LHb) and corelease both glutamate and GABA (Faget et al., 2018; Tooley et al., 2018). Since glutamate packaging into synaptic vesicles requires VGLUTs, synaptic glutamate release should not be affected by *Vgat* editing. Using this internal control, we can then normalize oIPSC amplitudes to optically evoked excitatory postsynaptic current (oEPSC) amplitudes to control for the number of terminals activated by optogenetic stimulation.

**Figure 2.**
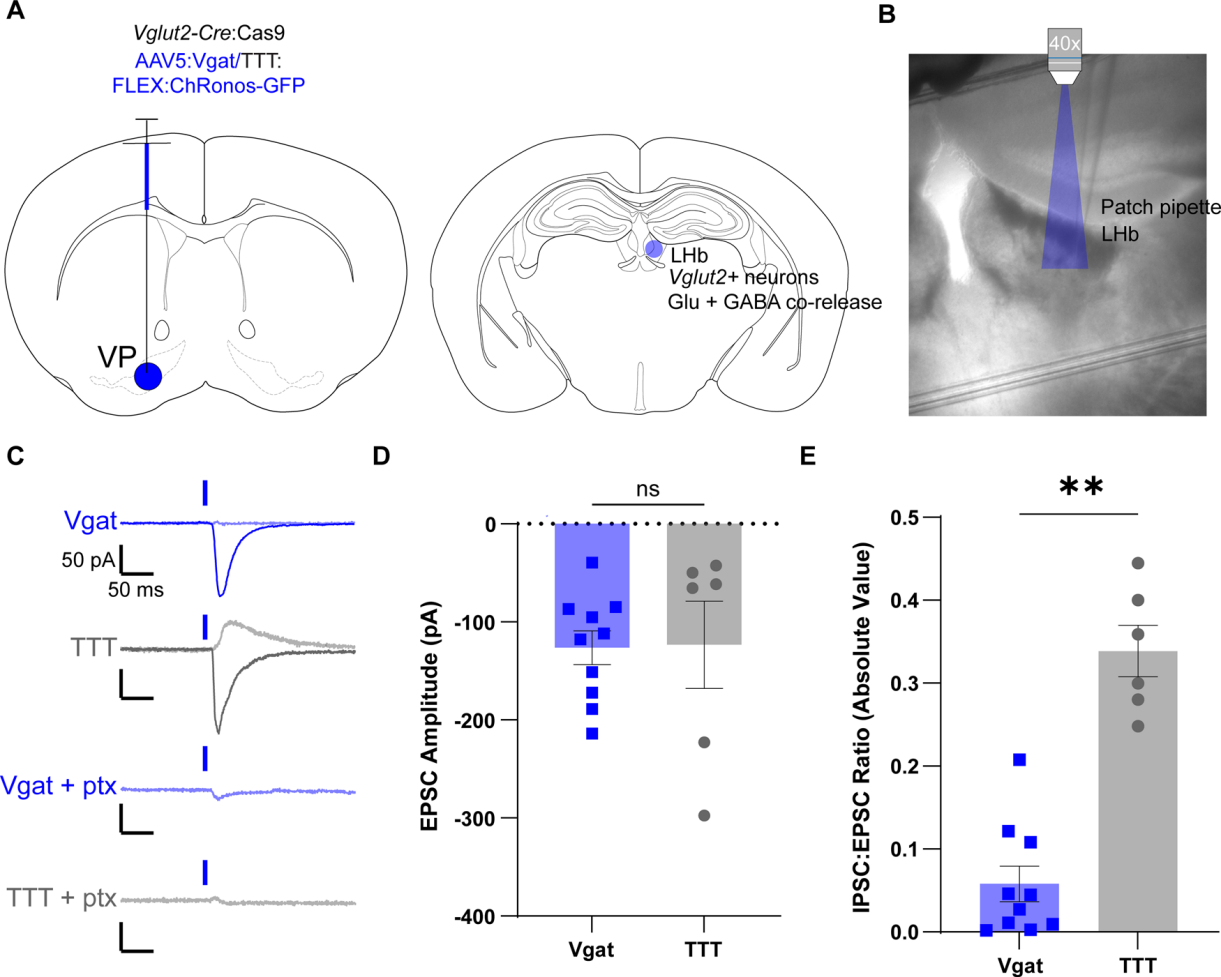
ChRonos-GFP coupled with *Vgat* editing selectively reduces inhibitory synaptic currents from GABA/Glutamate-coreleasing neurons. ***A***, Diagram of virus injection into the VP of 6–8-week-old *Vglut2-*Cre:Cas9 mice (left) and termination of *Vglut2*+ GABA/glutamate-coreleasing neurons from the VP in the LHb (right). ***B***, Representative 4× image of slice physiology recording in the LHb. The area of blue light stimulation of ChRonos shown in blue. ***C***, Representative EPSC (solid) and IPSC (shaded) traces for Vgat-edited (first row, blue) and TTT control (second row, gray) neurons and representative traces for Vgat-edited (third row, blue) and TTT control (fourth row, gray) oIPSC blocked with picrotoxin (ptx). Traces are aligned to light pulse onset (blue rectangle). The representative Vgat-edited oEPSC and oIPSC traces are an average of 49 consecutive sweeps each; the representative TTT control oEPSC and oIPSC traces are an average of 27 and 12 consecutive sweeps, respectively. The representative Vgat-edited oIPSC plus ptx is an average of 35 consecutive sweeps, and the representative control oIPSC plus ptx is an average of 15 consecutive sweeps. Horizontal scale bars = 50 ms; vertical scale bars = 50 pA. ***D***, Quantification of oEPSC amplitude for Vgat (blue) and TTT (gray) neurons. Each point represents the average evoked excitatory current amplitude from 10 ms before through 10 ms after the peak amplitude for 10–50 consecutive sweeps. The average Vgat oEPSC amplitude was −126.3 ± 17.12 pA (*n* = 10 neurons from 4 animals); the average TTT oEPSC amplitude was −123.3 ± 44.47 pA (*n* = 6 neurons from 3 animals; *p* = 0.94; ns^c^). ***E***, Quantification of the absolute value of the oIPSC:oEPSC ratio for Vgat-edited (left, blue) and TTT control (right, gray) neurons. oIPSC measurements were obtained by averaging the current values 10 ms before through 10 ms after the peak current amplitude. Each point represents the absolute value of the oIPSC 20 ms average divided by the oEPSC 20 ms average. The mean Vgat oIPSC:oEPSC ratio was 0.058 ± 0.021 (*n* = 10 neurons from 4 animals); the mean TTT oIPSC:oEPSC ratio was 0.34 ± 0.031 (*n* = 6 neurons from 3 animals; *p* = 0.0028**^d^). All comparisons were done using a two-tailed, nested *t* test. All data are reported as mean ± SEM.

We injected either the control (TTT) or *Vgat* targeted viruses described above into the VP of *Vglut2*-Cre:lsl-Cas9 mice (Fig. 2*A*). After 6 weeks, we performed slice electrophysiology recordings from neurons in the LHb while stimulating the VP → LHb projection terminals with blue light (1 ms, 1 mW/mm^2^, Fig. 2*B*). We recorded oEPSCs at −55 mV and oIPSCs at +10 mV in the same cell. As expected, oEPSC amplitudes were not significantly different between edited and control groups (Fig. 2*C,D*), suggesting similar efficacy of optogenetic activation with ChRonos between groups. However, we found a significant reduction in oIPSC:oEPSC ratios in edited animals compared with controls (Fig. 2*C,E*), and evoked outward currents were blocked with picrotoxin (Fig. 2*C*). This finding provides additional evidence for efficient *Vgat* editing in a different neuronal population with a robust internal control.

### *Grin1* editing in dopaminergic VTA neurons reduces synaptic NMDAR currents

**Figure 3.**
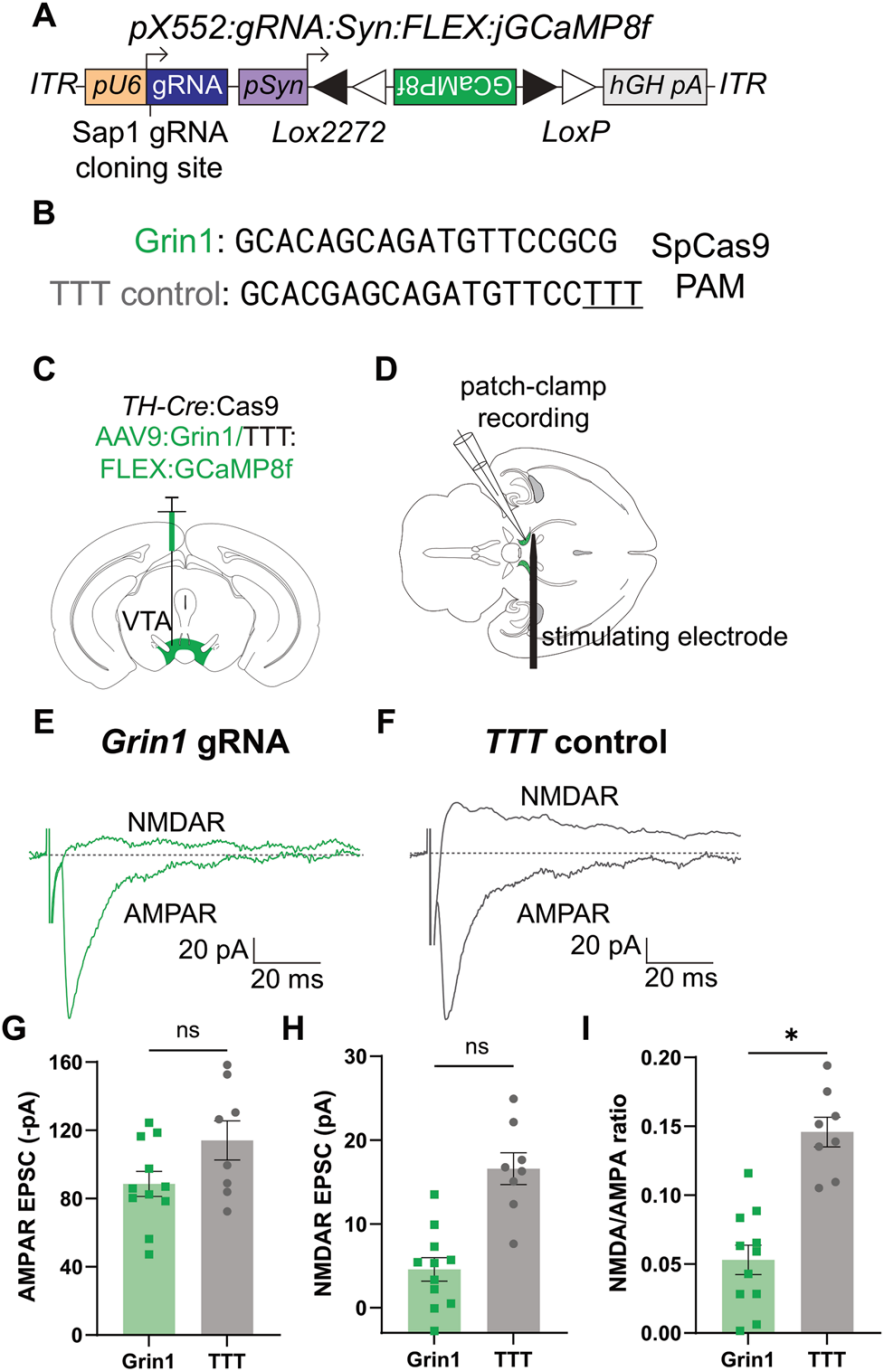
*Grin1* knockdown selectively reduces NMDAR current in VTA DA neurons. ***A***, Schematic of pX552 vector with Cre-dependent GCaMP8f transgene and Grin1 gRNA. ***B***, Sequences of gRNA targeting *Grin1* (top) and modified TTT control (bottom). ***C***, Diagram of virus injection into the VTA of 6–8-week *TH-*Cre:Cas9 mice. ***D***, Diagram of patch-clamp recording setup with recording electrode and stimulating electrode in the VTA. ***E***, Representative traces from Grin1-edited neurons of evoked NMDAR current (top) and AMPAR current (bottom). Traces are aligned to stimulating electrode pulse. Each trace represents a single trial. Horizontal scale bar = 20 ms; vertical scale bar = 20 pA. ***F***, Representative traces from TTT control neurons of evoked NMDAR current (top) and AMPAR current (bottom). Traces are aligned to stimulating electrode pulse. Each trace represents a single trial. Horizontal scale bar = 20 ms; vertical scale bar = 20 pA. ***G***, Quantification of AMPAR EPSC amplitude in Grin1 (green, left) and TTT (gray, right) neurons. Each point represents the average peak AMPAR current amplitude from 10–15 consecutive sweeps for one neuron. The average Grin1 peak AMPAR current was −88.61 ± 7.39 pA (*n* = 11 cells from 3 animals); the average TTT peak AMPAR current was −114.1 ± 11.47 pA (*n* = 8 cells from 3 animals; *p* = 0.12; ns^e^). ***H***, Quantification of NMDAR EPSC amplitude in Grin1 (green, left) and TTT (gray, right) neurons. Each point represents the average peak NMDAR current amplitude from 10–15 consecutive sweeps for one neuron. The average Grin1 peak NMDAR current was 4.58 ± 1.41 pA (*n* = 11 cells from 3 animals); the average TTT peak NMDAR current was 16.60 ± 1.90 pA (*n* = 8 cells from 3 animals; *p* = 0.052; ns^f^). ***I***, Quantification of NMDAR:AMPAR ratio in Grin1 (green, left) and TTT (gray, right) neurons. Each point represents the ratio of the average peak NMDAR current amplitude to the average peak AMPAR current amplitude from 10–15 consecutive sweeps for one neuron. The average Grin1 NMDAR:AMPAR ratio was 0.053 ± 0.011 (*n* = 11 cells from 3 animals); the average TTT NMDAR:AMPAR ratio was 0.146 ± 0.011 (*n* = 8 cells from 3 animals; *p* = 0.027*^g^). All comparisons were done using a two-tailed, nested *t* test. All data are reported as mean ± SEM. See Extended Data Figure 3-1 for more details.

Next, we tested whether we could use our approach to selectively record the activity of edited neurons using a fluorescent Ca^2+^ sensor. For these experiments, we targeted NMDA receptors (NMDARs) in dopaminergic neurons in the ventral tegmental area (VTA) due to robust NMDAR activation in response to rewarding stimuli (Overton and Clark, 1997; Stuber et al., 2008; Harnett et al., 2009; Zweifel et al., 2009; Paladini and Roeper, 2014). We created a viral vector containing Cre-dependent GCaMP8f (Zhang et al., 2023) using the same pX552 backbone as above (Fig. 3*A*). We then cloned in gRNA against the *Grin1* subunit of the NMDA receptor or a TTT control (Fig. 3*B*) and packaged these constructs into AAV9 serotyped viruses. To selectively express Cas9 in dopaminergic (DA) neurons, we crossed mice with Cre knocked into the tyrosine hydroxylase (*Th*) gene (Lindeberg et al., 2004) to a Cre-dependent Cas9 line (Chiou et al., 2015). We then injected 6–8-week-old adult TH-Cre:lsl-Cas9 mice from the F1 generation with control or active virus in the ventral tegmental area (VTA) to validate NMDAR knockdown using slice electrophysiology (Fig. 3*C*).

10.1523/ENEURO.0438-23.2024.f3-1 Figure 3-1 *Grin1* nonsense mediated decay in the VTA. (**A**) Representative *in situ* hybridization images for Grin1 (top) and TTT control (bottom) injections into the VTA of adult *TH-*Cre;igs-Cas9 mice. **Left:** 40x images staining for *Gcamp8f* mRNA. **Middle:** 40x images staining for *Grin1* mRNA. **Right:** Merged images showing *Gcamp8f* in green, *Grin1* in magenta, and DAPI in cyan. Scale bars=20µm. (**B**) Quantification of the area-normalized fluorescence intensity of *Grin1* mRNA transcripts in Grin1 edited vs. TTT control tissue. The average fluorescence intensity of the Grin1 group was 129.5±16.6 AU/µm^2^ (n=2771 cells from 3 animals); the average for the TTT control group was 115.0±36.9 AU/µm^2^ (n=3482 cells from 3 animals) (p=0.74; ns^w^). (**C**) Quantification of the number of *Grin1* mRNA puncta per µm^2^ in Grin1 edited vs. TTT control tissue. The average number of puncta for the Grin1 group was 0.11±0.01 puncta/µm^2^ (n=2771 cells from 3 animals); the average for the TTT control group was 0.11±0.004 puncta/µm^2^ (n=3482 cells from 3 animals) (p=0.53; ns^x^). (**D**) Quantification of the *Grin1* mRNA percent area per cell. The average %Area/Cell for the Grin1 group was 10.11±0.73% (n=2771 cells from 3 animals); the average for the TTT control group was 8.67±1.65% (n=3482 cells from 3 animals) (p=0.47; ns^y^). All comparisons were done using a 2-tailed, nested t-test. All data are reported as mean±SEM. Download Figure 3-1, TIF file.

After 6 weeks, we recorded from GCaMP8f+ VTA DA neurons in acute horizontal slices and measured electrically evoked AMPAR- and NMDAR-mediated EPSCs (Fig. 3*D*). Evoked AMPAR EPSCs, measured at −70 mV, were not significantly different between edited and control animals (Fig. 3*E–G*). While evoked NMDAR EPSCs in *Grin1*-edited neurons were not significantly reduced compared with controls (Fig. 3*H*), normalizing NMDAR EPSCs to AMPAR EPSCs revealed a strong and significant effect of editing in *Grin1* neurons compared with controls (Fig. 3*I*). These data demonstrate that we can efficiently suppress synaptic NMDAR function in VTA DA neurons by editing *Grin1* subunits.

To assess whether our construct induces NMD of mutated *Grin1* mRNA, we injected a separate cohort of mice with either active Grin1 virus or TTT control. After 6 weeks, we performed in situ hybridization for *Gcamp8f* and *Grin1* (Extended Data Fig. 3-1*A*). We failed to identify a significant reduction in the area-normalized *Grin1* fluorescence density, number of *Grin1* puncta/µm^2^, or *Grin1* puncta %Area per infected cell (Extended Data Fig. 3-1*B–D*), despite significant functional knockdown, which could be attributed to the longer first coding exon of this transcript and the proximity of the gRNA to the start codon (Lindeboom et al., 2016, 2019).

### In vivo calcium imaging in gene-edited VTA neurons

**Figure 4.**
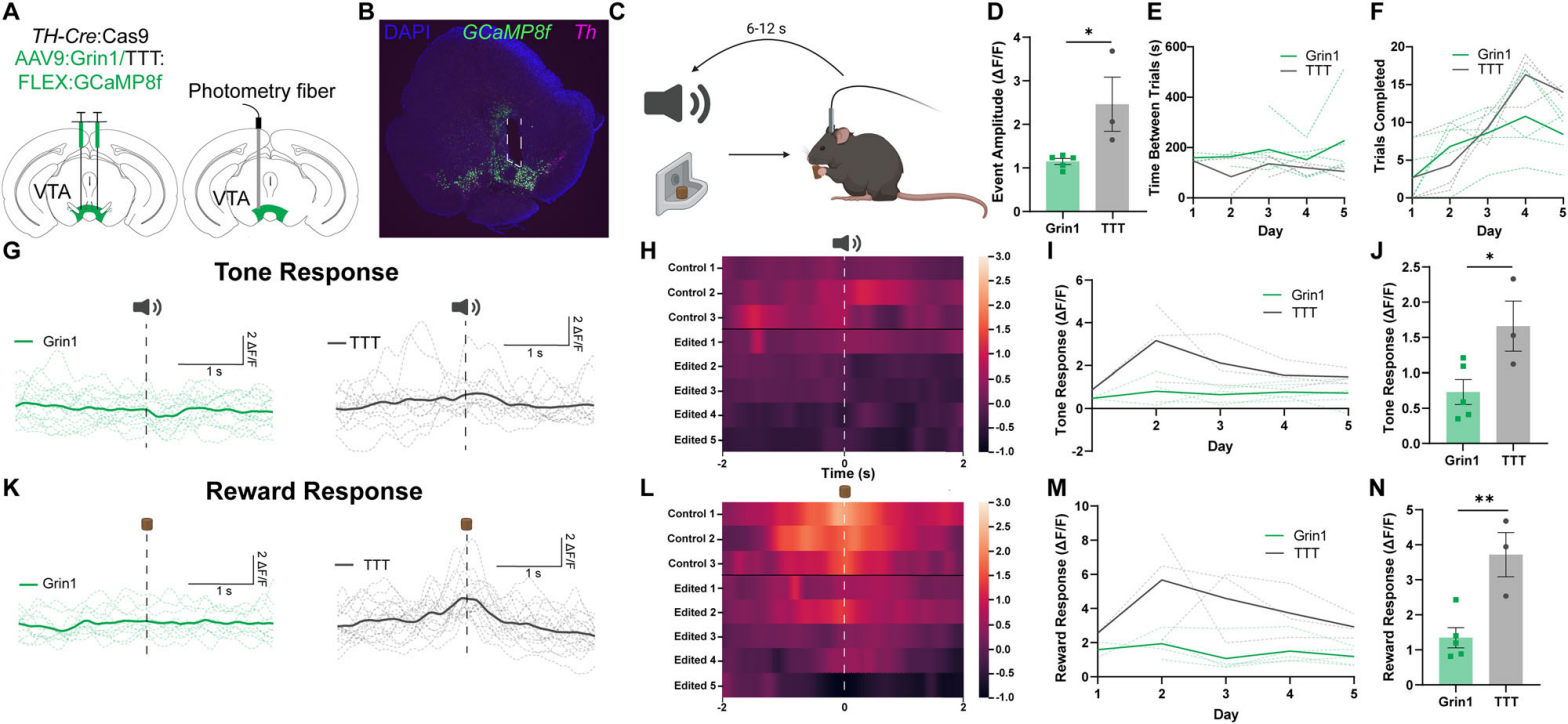
GCaMP8f Ca^2+^ currents are significantly reduced during a pavlovian reward task after *Grin1* knockdown in VTA DA neurons. ***A***, Diagram of bilateral virus injection (left) and unilateral fiber-optic insertion (right) into the VTA of 6–8-week *TH-*Cre:Cas9 mice. ***B***, Representative fluorescence ISH image of GCaMP8f virus expression in the VTA (green) overlapping with *TH*+ neurons (purple). The implant site indicated by white dashed lines. ***C***, Schematic of pavlovian cue/reward conditioning paradigm. ***D***, Quantification of all GCaMP8f transient amplitudes in Grin1 (green, left) and TTT (gray, right) conditions. Each point represents the average event amplitude, measured as a change in fluorescence over baseline (ΔF/F) across all recording sessions for one animal. The average Grin1 event amplitude was 1.15 ± 0.07; the average TTT event amplitude was 2.46 ± 0.62 (*p* = 0.029*^h^). ***E***, Quantification of time between trials for Grin1 (green) and TTT (gray) conditions across trial days. The dashed lines represent individual subjects; the green and gray solid lines represent the average for Grin1 and TTT conditions, respectively. Day 1, Due to insufficient data, a *t* test could not be performed on Day 1. Day 2, Grin1 average = 163.7 ± 12.8 s; TTT average = 84.3 ± 73.0 s (*p* = 0.47; ns). Day 3, Grin1 average = 192.1 ± 45.1 s; TTT average = 135.9 ± 26.8 s (*p* = 0.33; ns). Day 4, Grin1 average = 151.9 ± 30.5 s; TTT average = 119.4 ± 15.8 s (*p* = 0.38; ns). Day 5, Grin1 average = 227.7 ± 73.7 s; TTT average = 105.3 ± 17.8 s (*p* = 0.17; ns). These data were analyzed using an unpaired, two-tailed *t* test with Welch’s correction for unequal variances and corrected for multiple comparisons using the false discovery rate (FDR) approach, with an FDR threshold of 1%^i^. ***F***, Quantification of the average number of trials per day for Grin1 (green) and TTT (gray) conditions. The dashed lines represent individual subjects; the green and gray solid lines represent the average for Grin1 and TTT conditions, respectively. Day 1, Grin1 average = 2.60 ± 1.66 trials; TTT average = 2.67 ± 2.67 trials (*p* = 0.98; ns). Day 2, Grin1 average = 6.80 ± 1.83 trials; TTT average = 4.33 ± 2.85 trials (*p* = 0.51; ns). Day 3, Grin1 average = 8.60 ± 1.57 trials; TTT average = 9.33 ± 1.45 trials (*p* = 0.74; ns). Day 4, Grin1 average = 10.80 ± 2.63 trials; TTT average = 16.33 ± 2.19 trials (*p* = 0.16; ns). Day 5, Grin1 average = 8.40 ± 1.54 trials; TTT average = 14.00 ± 0.58 trials (*p* = 0.02; ns). These data were analyzed using an unpaired, two-tailed *t* test with Welch’s correction for unequal variances and corrected for multiple comparisons using the FDR approach, with an FDR threshold of 1%^j^. ***G***, Representative GCaMP8f response to tone cue in Grin1 (green, left) and TTT (gray, right) conditions. The dashed lines represent individual trials within a single session; the solid line represents the average of all trials for a single session. Dashed black line = tone cue onset. ***H***, Heat map displaying average GCaMP8f tone response across all trial days for all animals. Dashed white line = tone cue onset. ***I***, Average peak tone cue response on each recording day for Grin1 (green) and TTT (gray) conditions. Dashed lines = daily tone response averages for individual subjects; solid lines = tone response average for Grin1 (green) and TTT (gray). ***J***, Quantification of average peak tone response for Grin1 (green, left) and TTT (gray, right). Each point represents the average peak GCaMP8f tone response across all recording sessions for one animal. The average Grin1 peak GCaMP8f tone response was 0.73 ± 0.18; the average TTT peak GCaMP8f tone response was 1.66 ± 0.36 (*p* = 0.038*^k^). ***K***, Representative GCaMP8f response to reward in Grin1 (green, left) and TTT (gray, right) conditions. The dashed lines represent individual trials within a single session; the solid line represents the average of all trials for a single session. Dashed black line = reward retrieval. ***L***, Heat map displaying average GCaMP8f reward response across all trial days for all animals. Dashed white line = reward retrieval. ***M***, Average peak reward response on each recording day for Grin1 (green) and TTT (gray) conditions. Dashed lines = daily reward response averages for individual subjects; solid lines = reward response average for Grin1 (green) and TTT (gray). ***N***, Quantification of average peak reward response for Grin1 (green, left) and TTT (gray, right). Each point represents the average peak GCaMP8f reward response across all recording sessions for one animal. The average Grin1 peak GCaMP8f reward response was 1.35 ± 0.29; the average TTT peak GCaMP8f reward response was 3.72 ± 0.63 (*p* = 0.0085**^l^). *N* = 5 Grin1-edited animals; 3 TTT control animals. Unless otherwise noted, all comparisons were performed using an unpaired, two-tailed, nested *t* test, where each subcolumn represents one animal and each data point within the subcolumn represents an individual trial. All data are reported as mean ± SEM.

Having validated the functional efficacy of our *Grin1* knock-out using slice electrophysiology, we next combined this approach with fiber photometry to record Ca^2+^ signaling in awake, behaving animals during a conditioned pavlovian cue/reward task. To test whether *Grin1* editing could reduce stimulus-evoked GCaMP8f Ca^2+^ signal amplitude, we injected viruses containing both gRNA against either *Grin1* or a TTT control and Cre-dependent GCaMP8f, as above, bilaterally into the VTA of adult TH-Cre/lsl:Cas9 mice (Fig. 4*A*, left). We also implanted a photometry fiber above the left injection site (Fig. 4*A*, right). Fiber placement was validated using histology and in situ hybridization for *Th* and GCaMP8f (Fig. 4*B*). After 6 weeks, mice were acclimated to the recording chamber. After 3 d of acclimation, we recorded GCaMP8f Ca^2+^ transients in VTA dopaminergic neurons over a period of 5 d while the mice performed a pavlovian cue/reward task, in which a tone accompanied the delivery of a food reward (Fig. 4*C*).

We found that, independent of task stimuli, GCaMP8f Ca^2+^ transients in edited animals were nearly absent and significantly reduced compared with control (Fig. 4*D*). Upon analyzing task performance, we found that edited and control animals did not differ significantly in their time between trials across all trial days (Fig. 4*E*) or in the number of trials completed per day (Fig. 4*F*). While we are likely underpowered to detect small differences in task performance between control and edited groups, previous reports suggest that NMDARs in dopaminergic VTA neurons may not play a significant role in cue/reward association learning (Molina and Alvarez-Sabín, 2009; Parker et al., 2010).

GCaMP8f Ca^2+^ transients in response to both cue and reward were significantly reduced in edited animals compared with controls. Edited animals displayed minimal response to the tone cue (Fig. 4*G*), a trend that held across subjects (Fig. 4*H*) and trial days (Fig. 4*I*). Control animals, meanwhile, exhibited a modest Ca^2+^ fluorescence increase in response to the tone, and the overall average peak GCaMP8f signal in response to the tone was significantly reduced in edited animals compared with control (Fig. 4*J*). The GCaMP8f transients in response to food reward were similarly reduced in edited animals (Fig. 4*K*), which held across subjects (Fig. 4*L*) and trial days (Fig. 4*M*). Control animals, in contrast, displayed a robust food reward response. The overall average peak Ca^2+^ response to food reward was significantly reduced in edited animals compared with the control (Fig. 4*N*). These data show that our CRISPR/Cas9 system for gene knock-out can be used in combination with fiber photometry to selectively monitor activity in genetically edited neurons during behavioral tasks.

### Anatomical tracing and CRISPR/Cas9 editing in the peripheral nervous system using systemic viral vectors

So far, we have demonstrated the efficacy of our single-vector CRISPR/Cas9 system for gene knock-out in the central nervous system of adult mice. To broaden the applications of this tool, we next tested it in the peripheral nervous system of neonatal mice. Conditional knock-out of *Dicer* in parvalbumin (*PV*)-positive peripheral proprioceptor neurons results in axonal retraction from their downstream targets in the ventral horn of the spinal cord (Imai et al., 2016).

**Figure 5.**
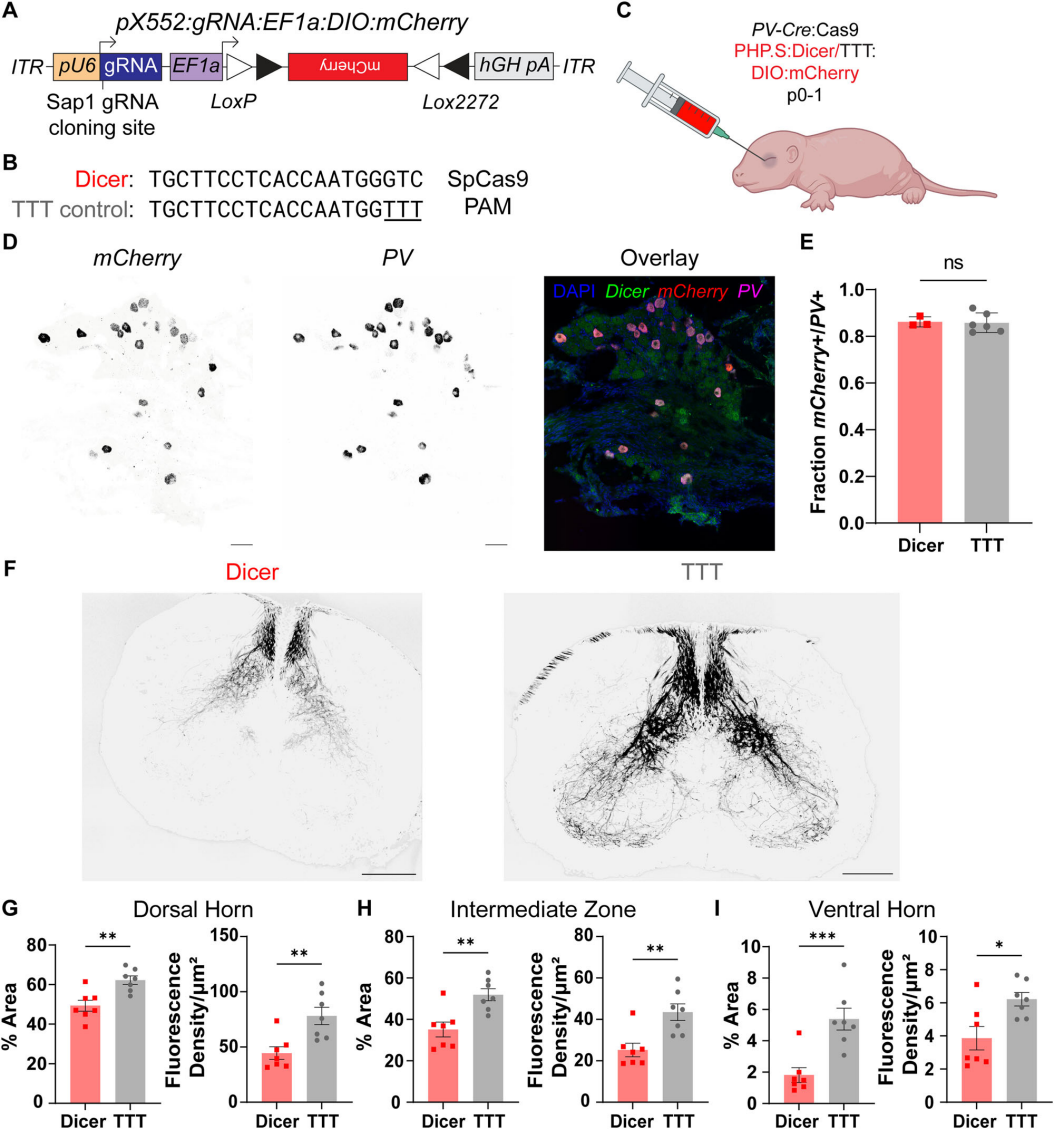
Cre-dependent mCherry expression and *Dicer* editing in peripheral PV+ neurons. ***A***, Schematic of pX552 vector with Cre-dependent mCherry transgene and Dicer gRNA. ***B***, Sequences of gRNA targeting *Dicer* (top) and modified TTT control (bottom). ***C***, Diagram of retro-orbital systemic virus injection into p0-1 *PV-*Cre:Cas9 mice. ***D***, 20× images of lumbar DRG from a p14 *PV-*Cre:Cas9 mouse labeled using ISH for *mCherry* (left) and *PV* (middle). Overlay (right) shows a high percentage of *mCherry*/*PV* overlap. Scale bars = 50 µm. ***E***, Quantification of *mCherry+*/*PV+* DRG neurons for Dicer (red, left) and TTT (gray, right). The average Dicer fraction of *PV+* cell bodies expressing *mCherry* was 0.87 ± 0.03 (*n* = 3 animals); the average TTT fraction was 0.85 ± 0.04 (*n* = 6 animals; *p* = 0.35; ns^m^). ***F***, 4× representative images of mCherry-expressing proprioceptive neuron processes in the spinal cord for Dicer (left) and TTT control (right) groups. Scale bar, 200 µm. ***G***, Quantification of %Area (left) and fluorescence density (right) of mCherry+ processes in the spinal cord dorsal horn in Dicer versus TTT groups. The average dorsal horn % fiber area for the Dicer group was 49.55 ± 2.61% (*n* = 123 slices from 7 animals); the average for the TTT control group was 62.31 ± 2.35% (*n* = 115 slices from 7 animals; *p* = 0.004**^n^). The average dorsal horn fluorescence density for the Dicer group was 44.80 ± 2.75 AU/µm^2^ (*n* = 123 slices from 7 animals); the average for the TTT group was 78.22 ± 5.18 AU/µm^2^ (*n* = 115 slices from 7 animals; *p* = 0.005**^o^). ***H***, Quantification of %Area (left) and fluorescence density (right) of mCherry+ processes in the spinal cord intermediate zone (IZ) in Dicer versus TTT groups. The average IZ % fiber area for the Dicer group was 34.60 ± 2.27% (*n* = 128 slices from 7 animals); the average for the TTT control group was 52.32 ± 1.94% (*n* = 116 slices from 7 animals; *p* = 0.002**^p^). The average IZ fluorescence density for the Dicer group was 25.28 ± 1.55 AU/µm^2^ (*n* = 128 slices from 7 animals); the average for the TTT group was 43.57 ± 2.13 AU/µm^2^ (*n* = 116 slices from 7 animals; *p* = 0.004**^q^). ***I***, Quantification of %Area (left) and fluorescence density (right) of mCherry+ processes in the spinal cord ventral horn in Dicer versus TTT groups. The average ventral horn % fiber area for the Dicer group was 1.84 ± 0.27% (*n* = 129 slices from 7 animals); the average for the TTT control group was 5.52 ± 0.44% (*n* = 116 slices from 7 animals; *p* = 0.001***^r^). The average ventral horn fluorescence density for the Dicer group was 3.83 ± 0.32 AU/µm^2^ (*n* = 129 slices from 7 animals); the average for the TTT group was 6.22 ± 0.32 AU/µm^2^ (*n* = 116 slices from 7 animals; *p* = 0.011*^s^). All comparisons were done using a two-tailed, nested *t* test. All data are reported as mean ± SEM. See Extended Data Figure 5-1 for more details.

To test whether these viral CRISPR/Cas9 approaches could be used to study genetic regulation of axonal morphology in the PNS, we first inserted a Cre-dependent mCherry transgene into the pX552 gRNA cloning vectors (Fig. 5*A*). We then developed gRNAs targeted against *Dicer* or a control gRNA with mismatch sequences adjacent to the SpCas9 PAM (Fig. 5*B*), cloned these into the pX552 mCherry vector, and packaged it into PNS-selective PHP.S serotyped AAVs (Chan et al., 2017). We injected active or control virus retro-orbitally in p0-1 *PV-*Cre/lsl-Cas9 mice to target PV+ proprioceptive neurons (Fig. 5*C*). After 14 d, we analyzed PV+ neuronal cell bodies in the DRG using ISH to determine the infection efficiency of the virus. Both edited and control viruses showed a high infection efficiency with no off-target expression (Fig. 5*D,E*). Analysis of mCherry+ spinal cord projections revealed a significant reduction in both fluorescence density and area innervated by mCherry+ spinal cord projections of PV+ neurons in edited compared with control mice in the dorsal, intermediate, and ventral aspects of the spinal cord (Fig. 5*F–I*). These findings recapitulate the phenotype seen with conditional Dicer knock-out in PV+ peripheral proprioceptive neurons, with a reduction in fiber density in both the ventral and dorsal horns of the spinal cord (Imai et al., 2016), and demonstrate that our single-vector CRISPR/Cas9 system can also be used at early postnatal stages of neural circuit development in the peripheral nervous system.

10.1523/ENEURO.0438-23.2024.f5-1 Figure 5-1 *Dicer* nonsense mediated decay in DRG neurons. (**A**) Representative *in situ* hybridization images for Dicer (top) and TTT control (bottom) expression in the DRG of p14 PV-Cre;igs-Cas9 mice. **Left:** 40x images staining for *mCherry* mRNA. **Middle:** 40x images staining for *Dicer* mRNA. **Right:** Merged images showing *mCherry* in red, *Dicer* in green, and DAPI in blue. Scale bars=20µm. (**B**) Quantification of the area-normalized fluorescence intensity of *Dicer* mRNA transcripts in Dicer edited vs. TTT control tissue. The average fluorescence intensity of the Dicer group was 131.0±6.4 AU/µm^2^ (n=1121 cells from 3 animals); the average for the TTT control group was 142.6±22.2 AU/µm^2^ (n=523 cells from 6 animals) (p=0.73; ns^z^). (**C**) Quantification of the number of *Dicer* mRNA puncta per µm^2^ in Dicer edited vs. TTT control tissue. The average number of puncta for the Dicer group was 0.02±0.003 puncta/µm^2^ (n=1121 cells from 3 animals); the average for the TTT control group was 0.02±0.004 puncta/µm^2^ (n=523 cells from 6 animals) (p=0.58; ns^aa^). (**D**) Quantification of the *Dicer* mRNA percent area per cell. The average %Area/Cell for the Dicer group was 1.10±0.30% (n=1121 cells from 3 animals); the average for the TTT control group was 1.42±0.37% (n=523 cells from 6 animals) (p=0.58; ns^bb^). All comparisons were done using a 2-tailed, nested t-test. All data are reported as mean±SEM. Download Figure 5-1, TIF file.

To assess whether our construct induces NMD of mutated *Dicer* mRNA, we performed in situ hybridization for *mCherry* and *Dicer* in the DRG of Dicer-edited and TTT control mice (Extended Data Fig. 5-1*A*). Despite demonstrating a reduction of mCherry+ axons in the spinal cords of edited animals, we failed to identify a significant reduction in the area-normalized *Dicer* fluorescence density, number of *Dicer* puncta/µm^2^, or *Dicer* puncta %Area per infected cell (Fig. 5-1*B–D*). Similar to our observations above, these data suggest that NMD may not be an accurate measure of editing efficiency for every gene.

## Discussion

These findings demonstrate the efficacy and broad utility of our single-vector approach for CRISPR/Cas9 gene editing and expression of genetically encoded tools. This method allows for efficient gene editing in both the central and peripheral nervous system and flexible expression of a variety of genetically encoded tools to permit in-depth interrogation of target genes in specific neural circuits and brain regions. When used with optogenetic or chemogenetic techniques, this method can be a powerful tool to study mechanisms of neurotransmitter release, learning, or synaptic plasticity. Combined with in vivo genetically encoded biosensor imaging, it can be used to understand how specific genes influence neural circuit dynamics. When expressed with fluorescent proteins, this tool can facilitate studies on the genetic drivers of neuronal morphology, connectivity, and survival. However, our approach has some limitations which should be taken into consideration.

Any leaky Cre expression during development will result in Cas9-positive cells that may not faithfully represent the intended cell populations in adult mice (Dietrich et al., 2000; Hébert and McConnell, 2000; Campsall et al., 2002). This should be tested with any newly developed Cre lines prior to using this approach. If this is the case, Cre-dependent Cas9 and gRNA vectors developed by the Zweifel Lab (Hunker et al., 2020) would be a more effective strategy. Alternatively, future iterations of this vector design could incorporate Cre-dependent U6-gRNA cassettes to circumvent the issue of leaky Cas9 expression and ensure editing only in the cells that express Cre at the desired developmental time point. Additionally, our approach is largely limited to mice where transgenic lines are more prevalent, although Cre-dependent Cas9 transgenic rats have recently been developed (Bäck et al., 2019).

CRISPR/Cas9 gene editing is also limited by the number and arrangement of transcript variants for the target gene. This approach works best when targeting a gene with one or a few transcript variants that all share at least one common exon, preferably early in its coding sequence (Shalem et al., 2014; Joung et al., 2017; Hunker et al., 2020). It is more challenging to effectively target genes for which there are many transcript variants with variable exon structures. Future improvements in this approach could involve introducing multiple gRNAs in the same vector, allowing for better targeting of all variants. By using Cre-dependent Cas9 mouse lines, rather than packaging Cas9 in the viral vector, there is ample space remaining in the virus for additional gRNAs to facilitate improved targeting of a single gene or targeting of multiple genes simultaneously.

Our strategy facilitates the endogenous protein-independent expression of genetic tools to study the effect of gene editing at the neuronal and circuit levels. Other CRISPR/Cas9 approaches exist to modify endogenous proteins with fluorescent proteins or antigen tags, which should be considered for questions involving the specific expression pattern and localization of the gene(s) of interest (Gao et al., 2019; Willems et al., 2020; Fang et al., 2021; Zhong et al., 2021).

We did not detect NMD of edited mRNA in any of the three genes studied in this paper. While NMD can be a valuable tool to suggest editing efficacy, it comes with many caveats that may explain why we did not see reduced mRNA expression despite validating the presence of indels (in the case of *Vgat*) and functional effects of gene editing in all genes. For instance, it is well-established that gRNAs in the last exon or 50 nt upstream of the last exon–exon junction are less effective at producing NMD (Nagy and Maquat, 1998; Le Hir, 2001; Kashima et al., 2006). In accordance with these rules, our gRNAs are exclusively located in the first common exon and never in the last or second-to-the-last exon. However, more recent analysis has revealed two additional “rules” for gRNA design: exons longer than 400 nt inhibit NMD, and guides within 150 nt of the start codon are less effective at producing NMD (Lindeboom et al., 2016, 2019). The first common exons for both *Vgat* and *Grin1* break the “long exon” rule, potentially explaining the lack of detectable NMD. As is evident from our paper and previous CRISPR approaches (Hunker et al., 2020), gRNAs in long exons can still produce robust indel frequencies and functional results. Further, all of our gRNA constructs were within 150 nt of the start codon. There is additional evidence to suggest that noncanonical translation initiation events and alternative mRNA splicing can circumvent NMD in up to 50% of cases (Tuladhar et al., 2019). Future users of this single-vector approach should design their gRNA targets with these rules and caveats in mind; however, we have demonstrated that even in the absence of detectable NMD using RNA ISH, we were able to achieve effective and efficient editing.

Any CRISPR/Cas9 editing approach may not completely knock out the target gene, rather, it functions more as a “knockdown.” In our slice electrophysiology recordings in the NAc, for example, we noted residual evoked IPSCs, even in areas of strong virus expression. This could be a result of the randomness in relying on NHEJ repair to introduce frameshift mutations, and some neurons may have retained at least one functional copy of the target gene (Canver et al., 2014; Hunker et al., 2020). This could also be due to the retention of existing VGAT that was produced prior to gene editing or nonspecific transport of GABA into vesicles via other solute transporters (Tritsch et al., 2012). These limitations highlight the importance of validating functional knockdown efficacy for each gene and model system.

In *PV*+ spinal cord projections of *Dicer*-edited mice, we were able to recapitulate the phenotype of reduced innervation seen with conditional Dicer knock-out (Imai et al., 2016). However, we noted a small population of persistent *PV+* projections in the ventral horn. This could again be due to incomplete editing of *Dicer* in all infected *PV*+ neurons. Alternatively, it is possible that some percentages of *PV*+ proprioceptive neurons have already become synaptically anchored in the ventral spinal cord by the time we injected the virus at p0-1 and thus were less susceptible to retraction due to *Dicer* editing. This possibility highlights one benefit of this approach over traditional conditional knock-out: the ability to precisely time editing to probe the role of genes of interest during development. This feature could be used to edit key genes involved in neuronal migration or synapse formation at multiple time points to better understand the precise developmental stages at which these genes function. Future studies could also use this gene editing approach in combination with in utero electroporation or viral transduction to achieve targeted gene editing at even earlier developmental time points.

This CRISPR/Cas9 approach could be used to more efficiently screen for the effects of editing gene candidates identified through clinical GWAS studies or transcriptomic studies from defined cell types in model organisms. Although not included here, these vectors could also be modified to express other Cre-dependent opto- or chemogenetic approaches to enable further mechanistic understanding of various genes throughout the nervous system. Our approach is also not limited to targeting neurons; in combination with the appropriate viral vectors and mouse lines, this tool could be used to manipulate genes in astrocytes or cells in other organs. Finally, this tool could be used to knockdown native gene expression and reintroduce a mutant gene variant in defined cell types, increasing its translational potential to study the impacts of mutations identified in clinical settings.

## Conclusion

In this study, we present a flexible, single-vector approach to CRISPR/Cas9 editing and expression of genetically encoded tools. We demonstrate its utility in various cell types and brain regions throughout the central nervous system and describe the first systemic CRISPR/Cas9 gene editing with coexpressed reporters in the PNS. By combining our single-vector approach with genetic tools commonly used in neuroscience—channelrhodopsins, GCaMP, and fluorescent proteins—we demonstrate its potential for flexible and precise interrogation of gene function in specific cell types and circuits throughout the nervous system.

## Data Availability

All data and analysis codes are available from the Lead Contact. All DNA and viral constructs generated here are deposited on Addgene and are also available upon request from the Lead Contact. Key materials can be found in the material availability table (Table 2).

**Table 2.** Statistical table

	Data structure	Type of test	Power
a	Normal distribution	Two-tailed, nested *t* test	95% CI of difference (Edited–control): −242.7 to −71.28 pA
b	Right-skewed distribution	Two-tailed, nested *t* test	95% CI of difference (Edited–control): −3,142 to −0.88 puncta
c	Normal distribution	Two-tailed, nested *t* test	95% CI of difference (Edited–control): −89.61 to 83.70 pA
d	Right-skewed distribution	Two-tailed, nested *t* test	95% CI of difference (Edited–control): −0.3928 to −0.1591
e	Normal distribution	Two-tailed, nested *t* test	95% CI of difference (Edited–control): −10.80 to 61.70 pA
f	Normal distribution	Two-tailed, nested *t* test	95% CI of difference (Edited–control): −21.34 to 0.1771 pA
g	Normal distribution	Two-tailed, nested *t* test	95% CI of difference (Edited–control): −0.1556 to −0.01637
h	Left-skewed distribution	Two-tailed unpaired nested *t* test	95% CI of difference (Edited–control): −2.437 to −0.1870
i	Right-skewed distribution	Two-tailed, unpaired *t* test with Welch’s correction for unequal variances and FDR correction for multiple comparisons	95% CI of difference (Edited–control): Day 2: −742.025 to 900.869s Day 3: −73.0067 to 185.3847s Day 4: −52.8448 to 117.9148s Day 5: −79.7831 to 324.7111s
j	Normal distribution	Two-tailed, unpaired *t* test with Welch’s correction for unequal variances and FDR correction for multiple comparisons	95% CI of difference (Edited–control): Day 1: −9.20 to 9.07 trials Day 2: −7.26 to 12.19 trials Day 3: −6.06 to 4.59 trials Day 4: −13.96 to 2.89 trials Day 5: −9.81 to −1.38 trials
k	Right-skewed distribution	Two-tailed unpaired nested *t* test	95% CI of difference (Edited–control): −1.851 to −0.073
l	Right-skewed distribution	Two-tailed unpaired nested *t* test	95% CI of difference (Edited–control): −3.836 to −0.8553
m	Normal distribution	Two-tailed, nested *t* test	95% CI of difference (Edited–control): −0.034 to 0.084
n	Normal distribution	Two-tailed, nested *t* test	95% CI of difference (Edited–control): −20.63 to −4.895%
o	Right-skewed distribution	Two-tailed, nested *t* test	95% CI of difference (Edited–control): −54.63 to −12.22 AU/µm^2^
p	Normal distribution	Two-tailed, nested *t* test	95% CI of difference (Edited–control): −27.62 to −7.82%
q	Normal distribution	Two-tailed, nested *t* test	95% CI of difference (Edited–control): −29.51 to −7.05 AU/µm^2^
r	Normal distribution	Two-tailed, nested *t* test	95% CI of difference (Edited–control): −5.53 to −1.82%
s	Right-skewed distribution	Two-tailed, nested *t* test	95% CI of difference (Edited–control): −4.13 to −0.65 AU/µm^2^
t	Normal distribution	Two-tailed, nested *t* test	95% CI of difference (Edited–control): −129.6 to 123.1 AU/µm^2^
u	Normal distribution	Two-tailed, nested *t* test	95% CI of difference (Edited–control): −0.03 to 0.09 puncta/ µm^2^
v	Normal distribution	Two-tailed, nested *t* test	95% CI of difference (Edited–control): −2.80 to 5.45%
w	Right-skewed distribution	Two-tailed, nested *t* test	95% CI of difference (Edited–control): −97.7 to 126.7 AU/µm^2^
x	Normal distribution	Two-tailed, nested *t* test	95% CI of difference (Edited–control): −0.01 to 0.02 puncta/ µm^2^
y	Right-skewed distribution	Two-tailed, nested *t* test	95% CI of difference (Edited–control): −3.56 to 6.44%
z	Right-skewed distribution	Two-tailed, nested *t* test	95% CI of difference (Edited–control): −88.0 to 64.8 AU/µm^2^
aa	Normal distribution	Two-tailed, nested *t* test	95% CI of difference (Edited–control): −0.02 to 0.01 puncta/ µm^2^
bb	Normal distribution	Two-tailed, nested *t* test	95% CI of difference (Edited–control): −1.60 to 0.97%

## Synthesis

Reviewing Editor: Sam Golden, The University of Washington Decisions are customarily a result of the Reviewing Editor and the peer reviewers coming together and discussing their recommendations until a consensus is reached. When revisions are invited, a fact-based synthesis statement explaining their decision and outlining what is needed to prepare a revision will be listed below. The following reviewer(s) agreed to reveal their identity: Larry Zweifel, Jocelyn Richard.

Dear Dr. Copits,

Your manuscript has been reviewed by two experts in the field. Overall, both reviewers found your manuscript to potentially be appropriate for publication in eNeuro pending revision and re-review. Both reviewers described your new method as a potentially useful tool to the field, especially for labs using transgenic mice, although it was also noted that the method has a potentially high barrier to adoption. Specific concerns relate to validation of off-target effects, proof of principle in specific cell populations, and statical analysis. Please see the full reviewer comments below, and if you with to revise, please address each point individually.

Reviewer 1:

In this manuscript, the authors introduce a method that allows for the simultaneous expression of a transgene, a stimulatory opsin (ChRonos) or a genetically encoded calcium indicator (GCaMP8f), along with a guide RNA for SpCas9-mediated gene mutagenesis. The authors claim that this provides advantages over existing single viral vector CRISPR/Cas9 systems by allowing for the use of a Cre-dependent SpCas9 mouse line which allows for the packaging of the guide with an alternative transgene. In general, the experiments are well designed, and the results are highly significant, in keeping with previously published methods for CRISPR/Cas9 mutagenesis in the mouse adult CNS. Because the method requires breeding the Cre-driver to the SpCas9 mouse line and given the fact that AAVs of the same serotype co-transduce with nearly 95% efficiency, the method does not appear significantly advantageous over existing methods. The doubled costs of maintaining two homozygous mouse lines to generate double heterozygotes to avoid ectopic recombination is likely to present a significant barrier to entry for some investigators. This added expense would be even greater for generating homozygous Cre-driver rats and homozygous Cas9 rats (Back et al., Neuron 2019). These caveats aside, the paper is well written and generally well executed, the strawman of the need for the method notwithstanding. With revision to address the lack of controls, the manuscript provides an additional set of tools for investigators and is likely suitable for publication in eNeuro.

Specific comments:

1. The authors do not provide any assessment of off-target effects. The targeted alleles and predicted off-targets should be assessed by targeted deep sequencing of these loci in the specific cell types. This will also provide additional information on mutagenesis efficacy.

2. It is now common practice for investigators to perform RNAscope analysis to assess nonsense-mediated mRNA decay. This provides an additional means to assess mutagenesis efficacy and should be performed for the targeted genes.

3. To truly establish the viability of the method, the investigators should demonstrate that mutagenesis of a gene in a specific cell type can result in a phenotypic change.

Reviewer 2:

The authors report the use of single viral vectors for the expression of both gRNAs and viral tools in mice expressing Cas9 in cre-positive neurons. They demonstrate the efficacy of this approach in knocking down Vgat in nucleus accumbens and ventral pallidum neurons while expressing ChRonos-GFP, knocking down Grin1 in VTA dopamine neurons while expressing GCaMP8f, and Dicer editing combined with mCherry expression in peripheral PV neurons. Overall this seems like a valuable addition to the CRISPR/Cas9 toolbox, especially for neuroscientists working with transgenic mice. There is some variability in the strength of the evidence for some of the scientific conclusions presented here, as well as some other minor issues described below.

1. Several issues with existing tools are mentioned in the Introduction, including transient activation or leaky expression by cre-driver promoters, time and costs associated with transgenic crosses, and lack of overlap between cells expressing gRNAs and genetic tools or reporters. The approach described here seems to most effectively address the latter issue, which does not receive enough emphasis in the introduction. Instead a substantial amount of space is spent leading up to the use of CRISPR/Cas9 systems, which doesn't seem necessarily here.

2. Statistical concerns: While nested analyses are used for photometry, they are not as far as I can tell for the electrophysiology and histological analyses when more than one sample (cells or slices) is assessed per subject. The conclusions would be more rigorous if they were based on statistical analyses that accounted for the lack of independence of these measurements.

3. I have some concerns about interpretation of the behavioral data during the GCaMP recordings from dopamine neurons during Pavlovian conditioning. The authors state that the Grin1 deletion did not have an effect on learning, but it isn't clear whether the control mice show learning in this experiment either. It is a somewhat unusual Pavlovian conditioning paradigm, in that the next trial is only initiated after reward is retrieved from the prior trial. Therefore, the data is presented as time between trials and average trials/day, but it is presented as an average across all training days. Did the control mice vary on these measures across days? There are hints of an effect even on these summed measured, though it is likely the experiment is underpowered to detect an effect. It also isn't clear that the design allows the animals to demonstrate behavioral responses to the cue itself, given the brief nature of the cue and the timing of the pellet delivery afterwards. More additional information on behavior over time, along with a potential tempering of the conclusions drawn, would improve the paper.

## Author Response

Reviewers' Comments:

Reviewer #1: In this manuscript, the authors introduce a method that allows for the simultaneous expression of a transgene, a stimulatory opsin (ChRonos) or a genetically encoded calcium indicator (GCaMP8f), along with a guide RNA for SpCas9-mediated gene mutagenesis. The authors claim that this provides advantages over existing single viral vector CRISPR/Cas9 systems by allowing for the use of a Cre-dependent SpCas9 mouse line which allows for the packaging of the guide with an alternative transgene. In general, the experiments are well designed, and the results are highly significant, in keeping with previously published methods for CRISPR/Cas9 mutagenesis in the mouse adult CNS. Because the method requires breeding the Cre-driver to the SpCas9 mouse line and given the fact that AAVs of the same serotype co-transduce with nearly 95% efficiency, the method does not appear significantly advantageous over existing methods. The doubled costs of maintaining two homozygous mouse lines to generate double heterozygotes to avoid ectopic recombination is likely to present a significant barrier to entry for some investigators. This added expense would be even greater for generating homozygous Cre-driver rats and homozygous Cas9 rats (Back et al., Neuron 2019). These caveats aside, the paper is well written and generally well executed, the strawman of the need for the method notwithstanding. With revision to address the lack of controls, the manuscript provides an additional set of tools for investigators and is likely suitable for publication in eNeuro.

We thank the reviewer for these comments and for their helpful suggestions below. With regards to the efficiency of AAV co-transduction, it is true that some brain regions (namely, the cortex) co-transduce with >90% efficiency (Chan et. al., 2017; Maturana et. al., 2023). However, this efficiency drops for non-cortical regions (Chan et. al., 2017) and is reduced even further for tissues outside the brain, e.g. the retina and cochlea (Akil, 2020; Palfi et. al., 2012). Furthermore, dual systemic injection of AAVs for co-transduction of the PNS is costly due to the high volume of virus required; viral volume is also a limiting factor in pups, which have limited circulatory volume.

While maintaining the transgenic crosses required here could add to the cost of this approach, the gene editing strategy in this manuscript may be advantageous for specific experiments requiring tight overlap of editing and transgene expression in regions with poor viral cotransduction. It is however more cost-effective than generating conditional knockout lines, which many labs commonly employ. We think that it is beneficial to have multiple options available so that groups can weigh the pros and cons of each, note and choose the best approach for their studies. We have expanded our discussion of both the pros and cons of this alternative single-virus approach in the introduction: "Injecting multiple viruses compounds inefficiencies in transduction, resulting in some edited neurons that are not labeled with the transgene, and some unedited neurons that nevertheless contain the transgene. While AAV co-infection in some brain regions is high, cotransduction efficiency is lower in the cerebellum and striatum (Chan et al., 2017; Juarez et al., 2023; Maturana et al., 2023). Co-labeling is also lower in tissues such as the retina and cochlea (Akil, 2020; Palfi et al., 2012). The mismatch between edited and transgene-expressing neurons could lead to data misinterpretations. Furthermore, systemic dual virus injection for editing in the peripheral nervous system (PNS) requires very high titers, making it prohibitively expensive. This approach is also limited by circulatory volume in young mice, severely curtailing the current utility of CRISPR/Cas9 gene editing with dual viruses in the PNS." Specific comments:

1. The authors do not provide any assessment of off-target effects. The targeted alleles and predicted off-targets should be assessed by targeted deep sequencing of these loci in the specific cell types. This will also provide additional information on mutagenesis efficacy.

We certainly agree with this important point and attempted to assess mutagenesis efficacy by dissociating cell bodies or isolating nuclei, FAC sorting, and sequencing from transduced tissues as in Hunker, et. al. (2020). However, we found that our NLS-tagged mRuby protein did not remain in the nucleus after either tissue dissociation or nuclei isolation. Thus we changed our strategy to rely on protein histology and functional readouts of our gene editing. However, we recognize the need to demonstrate mutagenesis and partnered with a genome editing core at our institution to transfect Neuro2a cells with our Vgat construct in vitro and sequence these cells to assess mutagenesis efficiency and off-target effects. Due to the 3-month turnaround for this revision, we opted to assess the activity of only our Vgat guide in this manner, as the main point of the paper was to offer a single-vector CRISPR approach, not the specific design or improvement of gRNAs targeting specific genes. We stress in the discussion that anyone using this (or any other CRISPR approach) should thoroughly validate gRNAs for their target genes before use. For off-target effects, we assessed our Vgat gRNA for activity at the top 2 sites located in exons as identified by CRISPOR: Onecut1 and Tmem119. The strategy of assessing off-target activity in exons, rather than via whole genome deep sequencing, as is common in the field of CRISPR/Cas9 gene editing (Hunker et. al., 2020; Swiech et. al., 2015). These data are reported in Figure 1-1A, Table 1-2, and in the results: "To validate the editing efficiency of our construct, we co-nucleofected Neuro2a cells with either our active Vgat plasmid or TTT control and a Cas9 expression vector. After 72 hours, cells underwent PCR and next-generation sequencing (NGS) at the cut site to identify the fraction of cells expressing NHEJ-mediated insertions or deletions. We found that the active Vgat construct had a mutagenesis efficiency of 69.9%; meanwhile, the TTT control plasmid had almost no activity (1.2%) at the indicated cut site (Figure 1-1A). We further tested whether our Vgat gRNA construct had off-target activity in other genes. We identified two potential off-target sites in the exons of Onecut1 and Tmem119 using CRISPOR (Concordet &Haeussler, 2018). We nucleofected Neuro2a cells as above and performed NGS at the off-target sites in Onecut1 and Tmem119. We found minimal off-target activity at each gene (Onecut1: 2.1%; Tmem119: 0.5%), indicating that our Vgat gRNA construct has minimal off-target activity in exons other than the target site (Table 1-2)." "Figure 1-1: Efficiency of NAc Vgat editing and nonsense-mediated decay. "(A) Quantification of the percentage of insertions, deletions, and single nucleotide polymorphisms (SNPs) in Neuro2a cells infected with active Vgat plasmid (top) or TTT control (bottom). In the Vgat sample, edits were present in 69.9% of reads, with deletions making up 41.8% of reads and insertions making up 27.7% of reads. SNPs were present in 0.4% of reads. The most common single edit was a +1 nucleotide insertion, which occurred in 556 of 2177 total reads (25.5%). In the TTT control group, edits were present in only 1.2% of reads, with SNPs making up 0.9% of reads and deletions making up 0.3% of reads. There were no insertions." "Table 1-2. Vgat gRNA off-target editing at 2 potential off-target sites located in the exons of Onecut1 and Tmem119." 2. It is now common practice for investigators to perform RNAscope analysis to assess nonsense-mediated mRNA decay. This provides an additional means to assess mutagenesis efficacy and should be performed for the targeted genes.

We thank the reviewer for their suggestion to look into NMD and performed RNAScope in situ hybridization to assess relative mRNA expression for all genes. Our thorough approach to NMD analysis revealed no significant reduction in fluorescence intensity/µm2, puncta/µm2, or puncta %Area for Vgat, Grin1, or Dicer1. At first, this result perplexed us, given the demonstration of NMD with previous gRNA constructs. However, upon further investigation we found situations under which NMD is inhibited (Kashima et. al., 2006; Le Hir et. al., 2001; Nagy &Maquat, 1998; Lindenboom et. al., 2016, 2019). Our Vgat and Grin1 guides both break the "long exon" rule, as they are located in exons >400nt long. All of our guides break the "150nt" rule, as they are within 150nt of the start codon. However, per Lindenboom (2019), guides that break these rules and fail to induce NMD can still induce effective protein knock-down and subsequent functional effects. Our paper bears this out, as does Hunker et. al. (2020), which uses a Grin1 gRNA that breaks both the "long exon" and "150nt" rules. We further found evidence that non-canonical translation initiation events and alternative mRNA splicing can circumvent NMD in up to 50% of cases (Tuladhar et. al., 2019). We report our NMD results in Figures 1-1B-E, 3-1, and 5-1, as well as in the appropriate results sections. We discuss best practices for gRNA design, as well as caveats of NMD, in the discussion. "Figure 1-1: Efficiency of NAc Vgat editing and nonsense-mediated decay. "(B) Representative in situ hybridization images for Vgat (top) and TTT control (bottom) injections into the nucleus accumbens of adult Vgat-Cre;igs-Cas9 mice. Left: 40x images staining for GFP mRNA. Middle: 40x images staining for Vgat mRNA. Right: Merged images showing GFP in green, Vgat in magenta, and DAPI in cyan. Scale bars=20µm. (C) Quantification of the area-normalized fluorescence intensity of Vgat mRNA transcripts in Vgat edited vs. TTT control tissue. The average fluorescence intensity of the Vgat group was 173.4{plus minus}25.9 AU/µm2 (n=4649 cells from 3 animals); the average for the TTT control group was 176.7{plus minus}37.3 AU/µm2 (n=4365 cells from 3 animals) (p=0.95; nst). (D) Quantification of the number of Vgat mRNA puncta per µm2 in Vgat edited vs. TTT control tissue. The average number of puncta for the Vgat group was 0.09{plus minus}0.02 puncta/µm2 (n=4649 cells from 3 animals); the average for the TTT control group was 0.07{plus minus}0.004 puncta/µm2 (n=4365 cells from 3 animals) (p=0.28; nsu). (E) Quantification of the Vgat mRNA percent area per cell. The average %Area/Cell for the Vgat group was 8.2{plus minus}1.5% (n=4649 cells from 3 animals); the average for the TTT control group was 6.9{plus minus}0.3% (n=4365 cells from 3 animals) (p=0.42; nsv). All comparisons were done using a 2-tailed, nested t-test. All data are reported as mean{plus minus}SEM." "Figure 3-1. Grin1 nonsense mediated decay in the VTA. (A) Representative in situ hybridization images for Grin1 (top) and TTT control (bottom) injections into the VTA of adult TH-Cre;igs-Cas9 mice. Left: 40x images staining for Gcamp8f mRNA. Middle: 40x images staining for Grin1 mRNA. Right: Merged images showing Gcamp8f in green, Grin1 in magenta, and DAPI in cyan. Scale bars=20µm. (B) Quantification of the area-normalized fluorescence intensity of Grin1 mRNA transcripts in Grin1 edited vs. TTT control tissue. The average fluorescence intensity of the Grin1 group was 129.5{plus minus}16.6 AU/µm2 (n=2771 cells from 3 animals); the average for the TTT control group was 115.0{plus minus}36.9 AU/µm2 (n=3482 cells from 3 animals) (p=0.74; nsw). (C) Quantification of the number of Grin1 mRNA puncta per µm2 in Grin1 edited vs. TTT control tissue. The average number of puncta for the Grin1 group was 0.11{plus minus}0.01 puncta/µm2 (n=2771 cells from 3 animals); the average for the TTT control group was 0.11{plus minus}0.004 puncta/µm2 (n=3482 cells from 3 animals) (p=0.53; nsx). (D) Quantification of the Grin1 mRNA percent area per cell. The average %Area/Cell for the Grin1 group was 10.11{plus minus}0.73% (n=2771 cells from 3 animals); the average for the TTT control group was 8.67{plus minus}1.65% (n=3482 cells from 3 animals) (p=0.47; nsy). All comparisons were done using a 2-tailed, nested t-test. All data are reported as mean{plus minus}SEM." "Figure 5-1. Dicer nonsense mediated decay in DRG neurons. (A) Representative in situ hybridization images for Dicer (top) and TTT control (bottom) expression in the DRG of p14 PV-Cre;igs-Cas9 mice. Left: 40x images staining for mCherry mRNA. Middle: 40x images staining for Dicer mRNA. Right: Merged images showing mCherry in red, Dicer in green, and DAPI in blue. Scale bars=20µm. (B) Quantification of the area-normalized fluorescence intensity of Dicer mRNA transcripts in Dicer edited vs. TTT control tissue. The average fluorescence intensity of the Dicer group was 131.0{plus minus}6.4 AU/µm2 (n=1121 cells from 3 animals); the average for the TTT control group was 142.6{plus minus}22.2 AU/µm2 (n=523 cells from 6 animals) (p=0.73; nsz). (C) Quantification of the number of Dicer mRNA puncta per µm2 in Dicer edited vs. TTT control tissue. The average number of puncta for the Dicer group was 0.02{plus minus}0.003 puncta/µm2 (n=1121 cells from 3 animals); the average for the TTT control group was 0.02{plus minus}0.004 puncta/µm2 (n=523 cells from 6 animals) (p=0.58; nsaa). (D) Quantification of the Dicer mRNA percent area per cell. The average %Area/Cell for the Dicer group was 1.10{plus minus}0.30% (n=1121 cells from 3 animals); the average for the TTT control group was 1.42{plus minus}0.37% (n=523 cells from 6 animals) (p=0.58; nsbb). All comparisons were done using a 2-tailed, nested t-test. All data are reported as mean{plus minus}SEM." Results (Vgat): "To assess whether our construct induces nonsense-mediated decay (NMD) of mutated Vgat mRNA, we injected either the active Vgat or TTT control virus into the NAc of 6-8 week Vgat-Cre/lsl-Cas9 mice. After 6 weeks, we performed in situ hybridization for Gfp and Vgat mRNA (Figure 1-1B). We failed to identify a significant reduction in the area-normalized Vgat fluorescence density, number of Vgat puncta/µm2, or Vgat puncta %Area per infected cell (Figure 1-1C-E)." Results (Grin1) "To assess whether our construct induces NMD of mutated Grin1 mRNA, we injected a separate cohort of mice with either active Grin1 virus or TTT control. 6 weeks later, we performed in situ hybridization for Gcamp8f and Grin1 (Figure 3-1A). We failed to identify a significant reduction in the area-normalized Grin1 fluorescence density, number of Grin1 puncta/µm2, or Grin1 puncta %Area per infected cell (Figure 3-1B-D), despite significant functional knockdown, which could be attributed to the longer first coding exon of this transcript and the proximity of the gRNA to the start codon (Lindeboom et al., 2016, 2019)." Results (Dicer) "To assess whether our construct induces NMD of mutated Dicer mRNA, we performed in situ hybridization for mCherry and Dicer in the DRG of Dicer-edited and TTT control mice (Figure 5-1A). Despite demonstrating a reduction of mCherry+ axons in the spinal cords of edited animals, we failed to identify a significant reduction in the area-normalized Dicer fluorescence density, number of Dicer puncta/µm2, or Dicer puncta %Area per infected cell (Figure 5-1B-D). Similar to our observations above, these data suggest that NMD may not be an accurate measure of editing efficiency for every gene." Discussion "We did not detect NMD of edited mRNA in any of the three genes studied in this paper. While NMD can be a valuable tool to suggest editing efficacy, it comes with many caveats that may explain why we did not see reduced mRNA expression despite validating the presence of indels (in the case of Vgat) and functional effects of gene editing in all genes. For instance, it is well-established that gRNAs in the last exon or 50nt upstream of the last exon-exon junction are less effective at producing NMD (Kashima et al., 2006; Le Hir, 2001; Nagy &Maquat, 1998). In accordance with these rules, our gRNAs are exclusively located in the first common exon and never in the last or second-to-last exon. However, more recent analysis has revealed two additional "rules" for gRNA design: exons longer than 400nt inhibit NMD, and guides within 150nt of the start codon are less effective at producing NMD (Lindeboom et al., 2016, 2019). The first common exons for both Vgat and Grin1 break the "long exon" rule, potentially explaining the lack of detectable NMD. As is evident from our paper and previous CRISPR approaches (Hunker et al., 2020), gRNAs in long exons can still produce robust indel frequencies and functional results. Further, all of our gRNA constructs were within 150nt of the start codon. There is additional evidence to suggest that non-canonical translation initiation events and alternative mRNA splicing can circumvent NMD in up to 50% of cases (Tuladhar et al., 2019). Future users of this single-vector approach should design their gRNA targets with these rules and caveats in mind; however, we have demonstrated that even in the absence of detectable NMD using RNA ISH, we were able to achieve effective and efficient editing." 3. To truly establish the viability of the method, the investigators should demonstrate that mutagenesis of a gene in a specific cell type can result in a phenotypic change.

While we have robustly demonstrated that we can mutate 3 different genes in 3 different cell types and produce phenotypic changes in neurotransmitter release, NMDAR receptor function, in vivo neuronal activity, and axonal morphology, we admittedly do not report a robust behavioral change as a result of any of our manipulations. We agree that future experiments utilizing this approach to yield a change in some behavioral metric will strengthen the support for the vectors we have developed, and CRISPR/Cas9 editing more broadly, but this was not the primary goal of this manuscript. Thus, we chose to test the utility of our strategy for cell-specific transgene expression in combination gene editing for optogenetic experiments, in vivo Ca2+ imaging, and morphology.

Reviewer #2: The authors report the use of single viral vectors for the expression of both gRNAs and viral tools in mice expressing Cas9 in cre-positive neurons. They demonstrate the efficacy of this approach in knocking down Vgat in nucleus accumbens and ventral pallidum neurons while expressing ChRonos-GFP, knocking down Grin1 in VTA dopamine neurons while expressing GCaMP8f, and Dicer editing combined with mCherry expression in peripheral PV neurons. Overall this seems like a valuable addition to the CRISPR/Cas9 toolbox, especially for neuroscientists working with transgenic mice. There is some variability in the strength of the evidence for some of the scientific conclusions presented here, as well as some other minor issues described below.

We thank the reviewer for their suggestions below and are encouraged at their assessment that this will be a valuable addition to the CRISPR/Cas9 toolbox. Indeed, other labs are already obtaining promising preliminary data using viruses we have distributed.

1. Several issues with existing tools are mentioned in the Introduction, including transient activation or leaky expression by cre-driver promoters, time and costs associated with transgenic crosses, and lack of overlap between cells expressing gRNAs and genetic tools or reporters. The approach described here seems to most effectively address the latter issue, which does not receive enough emphasis in the introduction. Instead a substantial amount of space is spent leading up to the use of CRISPR/Cas9 systems, which doesn't seem necessarily here.

We thank the reviewer for their suggestion to focus the introduction more specifically on the benefits of our approach over existing CRISPR/Cas9 tools, and have eliminated the discussion of global knockouts and Cre-lox approaches in favor of a more targeted discussion of the limitations of current CRISPR/Cas9 systems and how this approach addresses them.

Revised Introduction: "Advances in genetic targeting have revealed how specific genes influence normal and pathological nervous system functions. Combining these manipulations with genetically encoded tools provides powerful approaches to understand how specific genes regulate synaptic, circuit, and behavioral phenotypes. Fluorescent proteins permit visualization of cellular structures (Chen et al., 2017; Meltzer et al., 2023; Tashiro et al., 2006; Young et al., 2008; J.-H. Zhang et al., 2011; X. Zhang et al., 2022), while calcium sensors enable optical recording of neuronal activity (Brandner et al., 2023; McQuillan et al., 2022; Zhou et al., 2022). Opsins and chemogenetic tools can manipulate the activity of defined cell types to better understand their function (Garrido et al., 2022; Hadjas et al., 2020; Rapanelli et al., 2017; Stuber et al., 2010).

CRISPR/Cas9 gene editing has emerged as an effective method to knock out genes in a cell-type specific manner. It uses bacterial Cas9 proteins, which complex with short guide RNAs (gRNA) to target specific DNA regions and cause a double-stranded break (DSB) (Jiang &Doudna, 2017; Jinek et al., 2012; Kalamakis &Platt, 2023). DSB repair via non-homologous end joining (NHEJ) often results in frame-shifting mutations generating a premature stop codon. Successful CRISPR/Cas9 editing has been demonstrated for multiple genes across diverse brain regions and cell types using viral vectors containing gRNA and Cre-dependent Cas9 injected into Cre-driver mice (Castro et al., 2021; Fellinger et al., 2021; Gunduz-Cinar et al., 2023; Hunker et al., 2020; Li et al., 2022; Liu et al., 2022; Soden et al., 2023; Swiech et al., 2015). However, this strategy requires injection of two AAV vectors to achieve both editing and transgene expression. This is because Cas enzymes are large; only a few can be packaged into AAVs, which have a maximum capacity of ~4.7 kb (Challis et al., 2019; Dong et al., 1996; Grieger &Samulski, 2005; Hunker et al., 2020). Thus, a second vector carrying a transgene must be co-injected. Injecting multiple viruses compounds inefficiencies in transduction, resulting in some edited neurons that are not labeled with the transgene, and some unedited neurons that nevertheless contain the transgene. While AAV co-infection in some brain regions is high, cotransduction efficiency is lower in the cerebellum and striatum (Chan et al., 2017; Juarez et al., 2023; Maturana et al., 2023). Co-labeling is also lower in tissues such as the retina and cochlea (Akil, 2020; Palfi et al., 2012). The mismatch between edited and transgene-expressing neurons could lead to data misinterpretations. Furthermore, systemic dual virus injection for editing in the peripheral nervous system (PNS) requires very high titers, making it prohibitively expensive. This approach is also limited by circulatory volume in young mice, severely curtailing the current utility of CRISPR/Cas9 gene editing with dual viruses in the PNS.

We designed and validated an alternative approach that addresses these limitations of two-vector CRISPR/Cas9 techniques. We first modified viral vectors with gRNA insertion sites (Swiech et al., 2015) to co-express Cre-dependent transgenes to visualize, manipulate, and record target neurons. To circumvent the size limitations of AAVs, we crossed Cre-dependent Cas9 mouse lines (Chiou et al., 2015; Platt et al., 2014) to Cre-driver lines to enable cell-type specific Cas9 expression. As this method only uses a single viral vector, the edited population of neurons fully overlaps with transgene expression. We validated this approach with the optogenetic tool ChRonos, GCaMP8f for recording Ca2+ transients, and mCherry for anatomical tracing. This approach can be used to screen and test the function of novel genes for effects on synaptic transmission, circuit activity, or morphology with a single viral injection." 2. Statistical concerns: While nested analyses are used for photometry, they are not as far as I can tell for the electrophysiology and histological analyses when more than one sample (cells or slices) is assessed per subject. The conclusions would be more rigorous if they were based on statistical analyses that accounted for the lack of independence of these measurements.

We appreciate the reviewer's insight into how we can improve our statistical analyses to be more rigorous, and strongly agree that nested t-tests for all experiments would be the more appropriate approach. We have followed the reviewers' advice and adjusted our statistical analyses accordingly, and are pleased to report that the more rigorous approach still supports our conclusion.

Table 1. Statistical Table 3. I have some concerns about interpretation of the behavioral data during the GCaMP recordings from dopamine neurons during Pavlovian conditioning. The authors state that the Grin1 deletion did not have an effect on learning, but it isn't clear whether the control mice show learning in this experiment either. It is a somewhat unusual Pavlovian conditioning paradigm, in that the next trial is only initiated after reward is retrieved from the prior trial. Therefore, the data is presented as time between trials and average trials/day, but it is presented as an average across all training days. Did the control mice vary on these measures across days? There are hints of an effect even on these summed measured, though it is likely the experiment is underpowered to detect an effect. It also isn't clear that the design allows the animals to demonstrate behavioral responses to the cue itself, given the brief nature of the cue and the timing of the pellet delivery afterwards. More additional information on behavior over time, along with a potential tempering of the conclusions drawn, would improve the paper.

We appreciate the reviewer's comments on the behavioral paradigm and analysis. To more appropriately show how each cohort performed across trial days, we have replaced the graphs in Figure 4E-F with graphs showing the average and individual times between trials and trials completed for each day and have adjusted the results section accordingly. We agree that this better represents how the animals' performance changes with repeat exposure to the paradigm and could show differences in learning between groups. We also adjusted our statistical analysis to assess differences in groups across trial days by performing multiple 2-tailed t-tests with corrections for multiple comparisons. We failed to identify a significant difference in time between trials or trials completed on any of the trial days. Although we found p=0.02 for number of trials completed on Day 5, this finding did not survive correction for multiple comparisons with a FDR of 1%. We agree with the reviewer that there may be a trend towards an effect of Grin1 editing, but that we are a) likely underpowered to detect it, and b) would need to design a more robust cue-reward paradigm to more effectively test for differences in learning and memory. As such, we have tempered our conclusions in the results to reflect our uncertainty around the effect of our manipulation on learning.

Figure 4. GCaMP8f Ca2+ currents are significantly reduced during a Pavlovian reward task after Grin1 knockdown in VTA DA neurons. "(E) Quantification of time between trials for Grin1 (green) and TTT (grey) conditions across trial days. Dashed lines represent individual subjects; green and grey solid lines represent the average for Grin1 and TTT conditions, respectively. Day 1: Due to insufficient data, a t-test could not be performed on Day 1. Day 2: Grin1 average=163.7{plus minus}12.8 s; TTT average=84.3{plus minus}73.0 s (p=0.47; ns). Day 3: Grin1 average=192.1{plus minus}45.1 s; TTT average=135.9{plus minus}26.8 s (p=0.33; ns). Day 4: Grin1 average=151.9{plus minus}30.5 s; TTT average=119.4{plus minus}15.8 s (p=0.38; ns). Day 5: Grin1 average=227.7{plus minus}73.7 s; TTT average=105.3{plus minus}17.8 s (p=0.17; ns). These data were analyzed using an unpaired, 2-tailed t-test with Welch's correction for unequal variances, and corrected for multiple comparisons using the False Discovery Rate (FDR) approach, with an FDR threshold of 1%i. (F) Quantification of average number of trials per day for Grin1 (green) and TTT (grey) conditions. Dashed lines represent individual subjects; green and grey solid lines represent the average for Grin1 and TTT conditions, respectively. Day 1: Grin1 average=2.60{plus minus}1.66 trials; TTT average=2.67{plus minus}2.67 trials (p=0.98; ns). Day 2: Grin1 average=6.80{plus minus}1.83 trials; TTT average=4.33{plus minus}2.85 trials (p=0.51; ns). Day 3: Grin1 average=8.60{plus minus}1.57 trials; TTT average=9.33{plus minus}1.45 trials (p=0.74; ns). Day 4: Grin1 average=10.80{plus minus}2.63 trials; TTT average=16.33{plus minus}2.19 trials (p=0.16; ns). Day 5: Grin1 average=8.40{plus minus}1.54 trials; TTT average=14.00{plus minus}0.58 trials (p=0.02; ns). These data were analyzed using an unpaired, 2-tailed t-test with Welch's correction for unequal variances, and corrected for multiple comparisons using the FDR approach, with an FDR threshold of 1%j." Results "Upon analyzing task performance, we found that edited and control animals did not differ significantly in their time between trials across all trial days (Figure 4E), nor in number of trials completed per day (Figure 4F). While we are likely underpowered to detect small differences in task performance between control and edited groups, previous reports suggest that NMDARs in dopaminergic VTA neurons may not play a significant role in cue-reward association learning (Molina &Alvarez-Sabín, 2009; Parker et al., 2010)."
