# Supplementary figures and images for: Cell-Specific Single Viral Vector CRISPR/Cas9 Editing and Genetically Encoded Tool Delivery in the Central and Peripheral Nervous Systems
Source: eNeuro. 2024 Jul 3;11(7):ENEURO.0438-23.2024. doi: 10.1523/ENEURO.0438-23.2024 (PMC11228695; doi:10.1523/ENEURO.0438-23.2024)

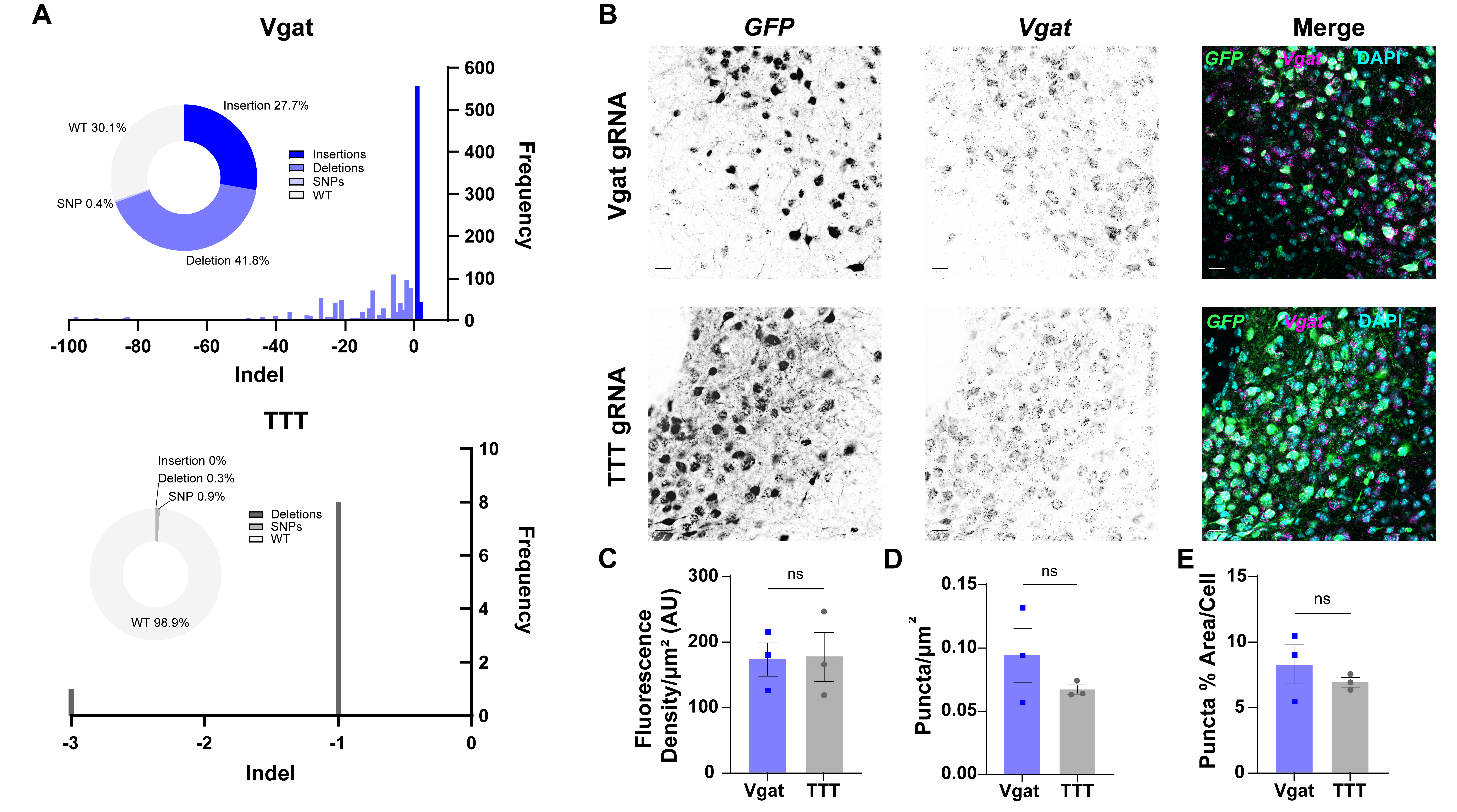

Supplement: Figure 1-1 — Efficiency of NAc Vgat editing and nonsense-mediated decay. (A) Quantification of the percentage of insertions, deletions, and single nucleotide polymorphisms (SNPs) in Neuro2a cells nucleofected with active Vgat plasmid (top) or TTT control (bottom). In the Vgat sample, edits were present in 69.9% of reads, with deletions making up 41.8% of reads and insertions making up 27.7% of reads. SNPs were present in 0.4% of reads. The most common single edit was a +1-nucleotide insertion, which occurred in 556 of 2177 total reads (25.5%). In the TTT control group, edits were present in only 1.2% of reads, with SNPs making up 0.9% of reads and deletions making up 0.3% of reads. There were no insertions. (B) Representative in situ hybridization images for Vgat (top) and TTT control (bottom) injections into the nucleus accumbens of adult Vgat-Cre;igs-Cas9 mice. Left: 40x images staining for GFP mRNA. Middle: 40x images staining for Vgat mRNA. Right: Merged images showing GFP in green, Vgat in magenta, and DAPI in cyan. Scale bars=20µm. (C) Quantification of the area-normalized fluorescence intensity of Vgat mRNA transcripts in Vgat edited vs. TTT control tissue. The average fluorescence intensity of the Vgat group was 173.4±25.9 AU/µm2 (n=4649 cells from 3 animals); the average for the TTT control group was 176.7±37.3 AU/µm2 (n=4365 cells from 3 animals) (p=0.95; nst). (D) Quantification of the number of Vgat mRNA puncta per µm2 in Vgat edited vs. TTT control tissue. The average number of puncta for the Vgat group was 0.09±0.02 puncta/µm2 (n=4649 cells from 3 animals); the average for the TTT control group was 0.07±0.004 puncta/µm2 (n=4365 cells from 3 animals) (p=0.28; nsu). (E) Quantification of the Vgat mRNA percent area per cell. The average %Area/Cell for the Vgat group was 8.2±1.5% (n=4649 cells from 3 animals); the average for the TTT control group was 6.9±0.3% (n=4365 cells from 3 animals) (p=0.42; nsv). All comparisons were done using a 2-tailed, nested t-test. All da [file eneuro-11-ENEURO.0438-23.2024-s002.tif]

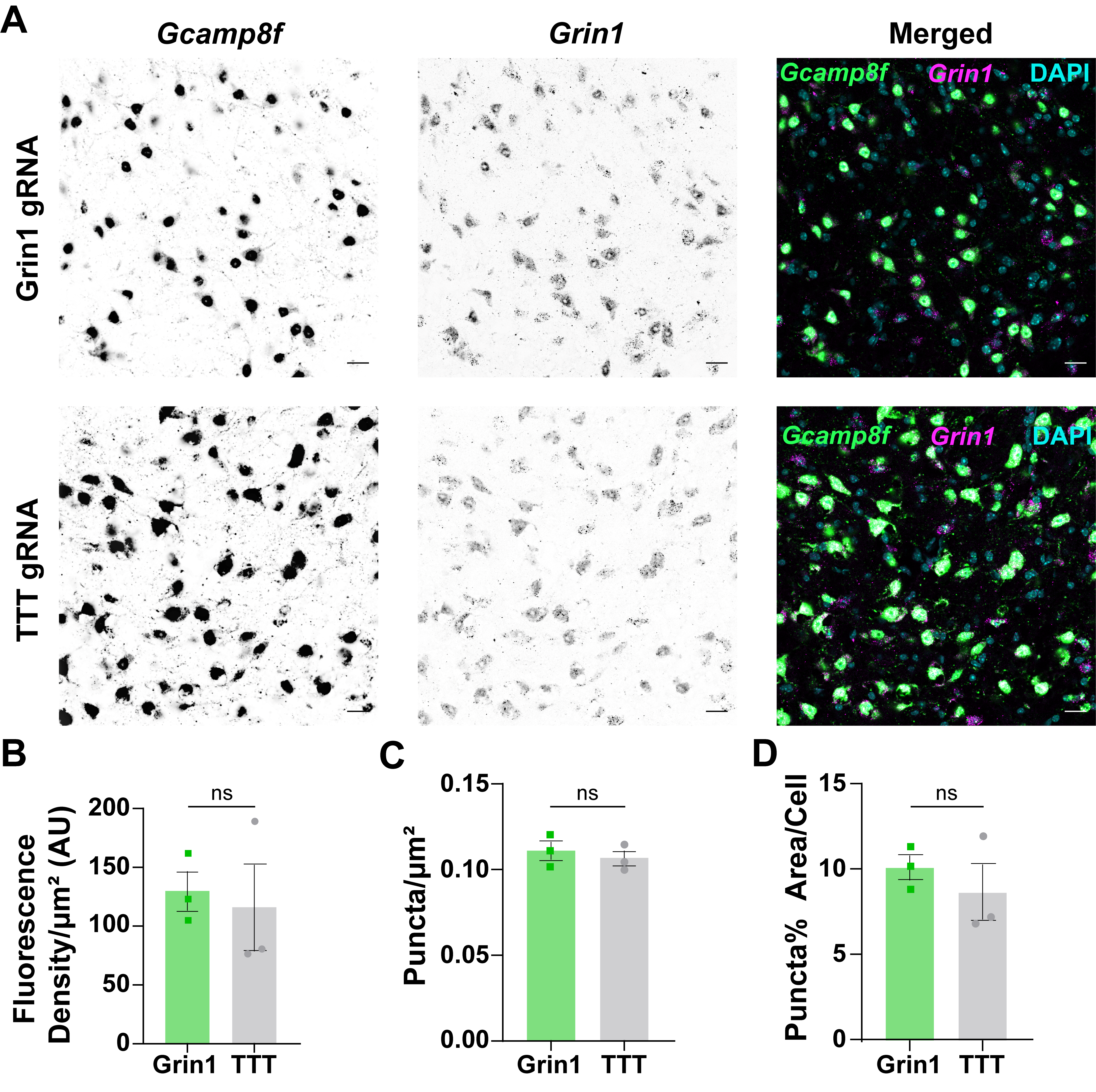

Supplement: Figure 3-1 — Grin1 nonsense mediated decay in the VTA. (A) Representative in situ hybridization images for Grin1 (top) and TTT control (bottom) injections into the VTA of adult TH-Cre;igs-Cas9 mice. Left: 40x images staining for Gcamp8f mRNA. Middle: 40x images staining for Grin1 mRNA. Right: Merged images showing Gcamp8f in green, Grin1 in magenta, and DAPI in cyan. Scale bars=20µm. (B) Quantification of the area-normalized fluorescence intensity of Grin1 mRNA transcripts in Grin1 edited vs. TTT control tissue. The average fluorescence intensity of the Grin1 group was 129.5±16.6 AU/µm2 (n=2771 cells from 3 animals); the average for the TTT control group was 115.0±36.9 AU/µm2 (n=3482 cells from 3 animals) (p=0.74; nsw). (C) Quantification of the number of Grin1 mRNA puncta per µm2 in Grin1 edited vs. TTT control tissue. The average number of puncta for the Grin1 group was 0.11±0.01 puncta/µm2 (n=2771 cells from 3 animals); the average for the TTT control group was 0.11±0.004 puncta/µm2 (n=3482 cells from 3 animals) (p=0.53; nsx). (D) Quantification of the Grin1 mRNA percent area per cell. The average %Area/Cell for the Grin1 group was 10.11±0.73% (n=2771 cells from 3 animals); the average for the TTT control group was 8.67±1.65% (n=3482 cells from 3 animals) (p=0.47; nsy). All comparisons were done using a 2-tailed, nested t-test. All data are reported as mean±SEM. Download Figure 3-1, TIF file. [file eneuro-11-ENEURO.0438-23.2024-s004.tif]
